# A role for the Gram-negative outer membrane in bacterial shape determination

**DOI:** 10.1073/pnas.2301987120

**Published:** 2023-08-22

**Authors:** Elayne M. Fivenson, Patricia D. A. Rohs, Andrea Vettiger, Marios F. Sardis, Grasiela Torres, Alison Forchoh, Thomas G. Bernhardt

**Affiliations:** ^a^Department of Microbiology, Blavatnik Institute, Harvard Medical School, Boston, MA 02115; ^b^HHMI, Chevy Chase, MD 20815

**Keywords:** peptidoglycan, lipopolysaccharide, membrane, morphogenesis, cell envelope

## Abstract

The cell envelope of Gram-negative bacteria consists of three distinct layers: the cytoplasmic membrane, a cell wall made of peptidoglycan (PG), and an asymmetric outer membrane (OM) composed of phospholipid in the inner leaflet and lipopolysaccharide (LPS) glycolipid in the outer leaflet. The PG layer has long been thought to be the major structural component of the envelope protecting cells from osmotic lysis and providing them with their characteristic shape. In recent years, the OM has also been shown to be a load-bearing layer of the cell surface that fortifies cells against internal turgor pressure. However, whether the OM also plays a role in morphogenesis has remained unclear. Here, we report that changes in LPS synthesis or modification predicted to strengthen the OM can suppress the growth and shape defects of *Escherichia coli* mutants with reduced activity in a conserved PG synthesis machine called the Rod complex (elongasome) that is responsible for cell elongation and shape determination. Evidence is presented that OM fortification in the shape mutants restores the ability of MreB cytoskeletal filaments to properly orient the synthesis of new cell wall material by the Rod complex. Our results are therefore consistent with a role for the OM in the propagation of rod shape during growth in addition to its well-known function as a diffusion barrier promoting the intrinsic antibiotic resistance of Gram-negative bacteria.

## Significance

The cell wall has traditionally been thought to be the main structural determinant of the bacterial cell envelope that resists internal turgor and determines cell shape. However, the outer membrane (OM) has recently been shown to contribute to the mechanical strength of Gram-negative bacterial envelopes. Here, we demonstrate that changes to OM composition predicted to increase its load-bearing capacity rescue the growth and shape defects of *Escherichia coli* mutants defective in the major cell wall synthesis machinery that determines rod shape. Our results therefore reveal a previously unappreciated role for the OM in bacterial shape determination in addition to its well-known function as a diffusion barrier that protects Gram-negative bacteria from external insults like antibiotics.

Gram-negative bacteria have a characteristic three-layered cell envelope comprised of an inner (cytoplasmic) membrane (IM), a relatively thin cell wall made of peptidoglycan (PG), and an outer membrane (OM). The OM bilayer is asymmetric with phospholipids in the inner leaflet and the lipopolysaccharide (LPS) glycolipid in the outer leaflet. For many years, the PG layer was thought to be the sole load-bearing component of the envelope with the OM primarily serving to protect Gram-negative cells from external insults like antibiotics (1, 2). However, it has recently become clear that in addition to providing a barrier function, the OM can also help cells resist internal turgor pressure (3). What has remained unknown is whether the OM also partners with the PG layer to define cell shape. Here, we report a genetic analysis of PG synthesis and cell shape determination that supports such a role for the OM.

The PG heteropolymer is composed of glycan chains with alternating units of N-acetylglucosamine (GlcNAc) and N-acetylmuramic acid (MurNAc) (4). A short peptide is attached to the MurNAc sugar and is used to cross-link adjacent glycans to form the cell wall matrix. Glycosyltransferases catalyze the polymerization of glycan polymers, whereas transpeptidases perform the cross-linking reaction. There are two major classes of PG synthases: class A Penicillin Binding Proteins (aPBPs) and complexes formed between SEDS (Shape, Elongation, Division, Sporulation) proteins and class B PBPs (bPBPs) (1, 2, 5). The aPBPs have both enzymatic functions in a single polypeptide, whereas in the SEDS-bPBP complexes, the SEDS protein promotes glycan polymerization and the bPBP provides the cross-linking activity (6–9).

The SEDS-bPBP complexes RodA-PBP2 (6–8, 10) and FtsW-FtsI (9) play essential roles in rod shape determination and cell division, respectively. In both cases, these synthases are part of larger multiprotein assemblies involving cytoskeletal filaments. The rod shape–determining system is called the Rod complex (a.k.a. the elongasome). It promotes the elongation of bacilli and maintains their characteristic rod shape. In addition to RodA-PBP2, the complex includes filaments of the actin-like MreB protein along with three membrane proteins of poorly understood function: MreC, MreD, and RodZ (11–18). The Rod complex has been observed to dynamically rotate around the long axis of the cell as it deposits new PG material to promote cell elongation. PG synthesis is required for the motion and MreB filaments are thought to orient it orthogonally to the long cell axis via a rudder-like mechanism (1, 7, 19–22).

To better understand the Rod complex function, we previously identified nonfunctional variants of MreC in *Escherichia coli* and selected for suppressor mutations that overcame their shape and viability defects (10, 23). One major class of suppressors encoded hypermorphic variants of PBP2 and RodA that provided important insight into the mechanism of Rod complex activation and the regulation of SEDS proteins (10). Genetic, structural, and cytological evidence suggests that MreC activates the complex by inducing a conformational change in PBP2, which in turn activates RodA, shifting the complex from an inactive to an active state (10). The role of MreD in the complex is not clear (23, 24). The signals that promote Rod complex activation also remain unknown, but the mechanism may involve the recognition of landmarks in the PG matrix by PBP2 (25).

In this report, we study a class of suppressors that restore the growth and shape of *mreC* hypomorphs. Instead of activating the Rod complex directly, these suppressors function by increasing the production of LPS. Further analysis of the suppression mechanism revealed that Rod complex mutants are impaired for LPS production. Additionally, we found that modifications to LPS predicted to stiffen the OM restore rod shape in cells defective for MreC by promoting the feedback mechanism via which MreB orients PG synthesis. Thus, our results suggest a potential connection between Rod complex activity and LPS synthesis and argue for a morphogenic role for the OM.

## Results

### Increased LPS Synthesis Suppresses a Rod Complex Defect.

**Fig. 1.**
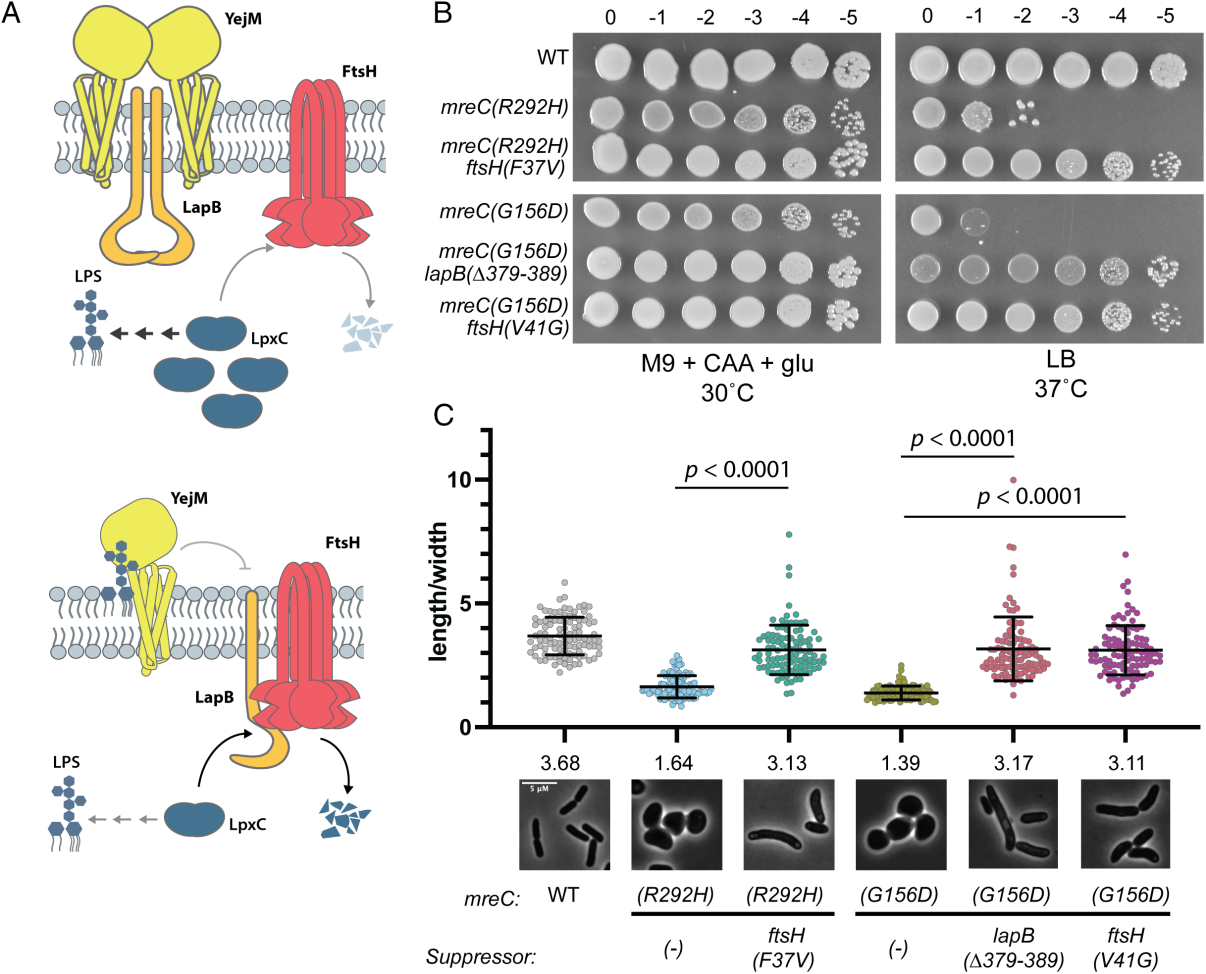
Mutations in factors involved in LpxC turnover rescue *mreC* hypomorphs. (*A*) Schematic overview of LpxC regulation. *Top*: When LPS levels are low, YejM interacts with LapB, sequestering it from the FtsH protease, leading to the stabilization of LpxC and increased LPS synthesis. *Bottom*: When LPS levels are high, LPS accumulates in the outer leaflet of the inner membrane. YejM binds to LPS, allowing LapB to interact with FtsH and target LpxC for degradation, reducing LPS synthesis. (*B*) WT (HC555), *mreC(R292H)* (PR5), *mreC(R292H) ftsH(V37G)* (PR82), *mreC(G156D)* (PR30), *mreC(G156D) lapB(∆379-389)* (PR86), *mreC(G156D) ftsH(V41G)* (PR88) were cultured for 24 h in minimal medium (M9 + CAA + glu) at 30 °C. Cultures were then normalized to OD_600_ = 1 and serially diluted and spotted onto LB and M9 + CAA + glu plates. LB plates were incubated for 16 h at 37 °C and M9 plates were incubated for 40 h at 30 °C. Dilution factors are indicated above the spot dilutions. (*C*) Micrographs of WT (HC555), *mreC(R292H)* (PR5), *mreC(R292H) ftsH(V37G)* (PR82), *mreC(G156D)* (PR30), *mreC(G156D) lapB(∆379-389)* (PR86), *mreC(G156D) ftsH(V41G)* (PR88). Strains were grown overnight in minimal medium (M9 + CAA + glu) at 30 °C. Overnight cultures were then back diluted to OD_600_ = 0.05 in minimal medium and incubated shaking at 30 °C until OD_600_ = 0.3-0.4. Cells were then spun down and resuspended in LB to an OD_600_ of 0.025 and incubated at 37 °C until OD_600_ = 0.3-0.4. Cells were then fixed and imaged. Aspect ratios were analyzed using the FIJI plugin MicrobeJ (39). (Scale bar, 5 µm.) n= 100 cells per group. Statistical significance determined using an unpaired *t* test with Welch’s correction (not assuming equal SDs).

Cells with *mreC(R292H)* or *mreC(G156D)* mutations produce stable MreC protein capable of inducing a dominant-negative growth and shape phenotype (10, 23). Therefore, the altered proteins are likely capable of joining the Rod complex but are defective in stimulating its activity. Mutants with these alleles at the native locus as their sole copy of *mreC* can be maintained as spheres on minimal medium (M9), but they fail to grow on rich medium (LB). We selected for spontaneous suppressors that restored the growth of these mutants on LB along with their rod shape. In addition to mutants encoding altered PBP2 and RodA described previously (10), the selection also identified suppressors in the *ftsH* and *lapB*(*yciM*) genes encoding regulators of LPS synthesis (Fig. 1 and *SI Appendix*, Table S1). FtsH is an IM metalloprotease that along with its adapter protein LapB (26, 27) degrades LpxC (UDP-3-O-acyl-N-acetylglucosamine deacetylase) (28–30), the enzyme that catalyzes the first committed step in LPS synthesis (31, 32). Proteolysis of LpxC is in turn regulated by the essential inner membrane protein YejM (PbgA, LapC), which functions to inhibit LapB activity in a manner that is sensitive to the concentration of LPS in the IM (30, 33–38). When the steady-state concentration is low due to LPS synthesis being balanced with its transport to the OM, YejM blocks LpxC turnover (Fig. 1 *A*, *Top*). However, when LPS synthesis outpaces its transport, YejM is inhibited by the buildup of LPS in the inner membrane and LpxC turnover is increased to restore homeostasis (Fig. 1 *A*, *Bottom*).

**Fig. 2.**
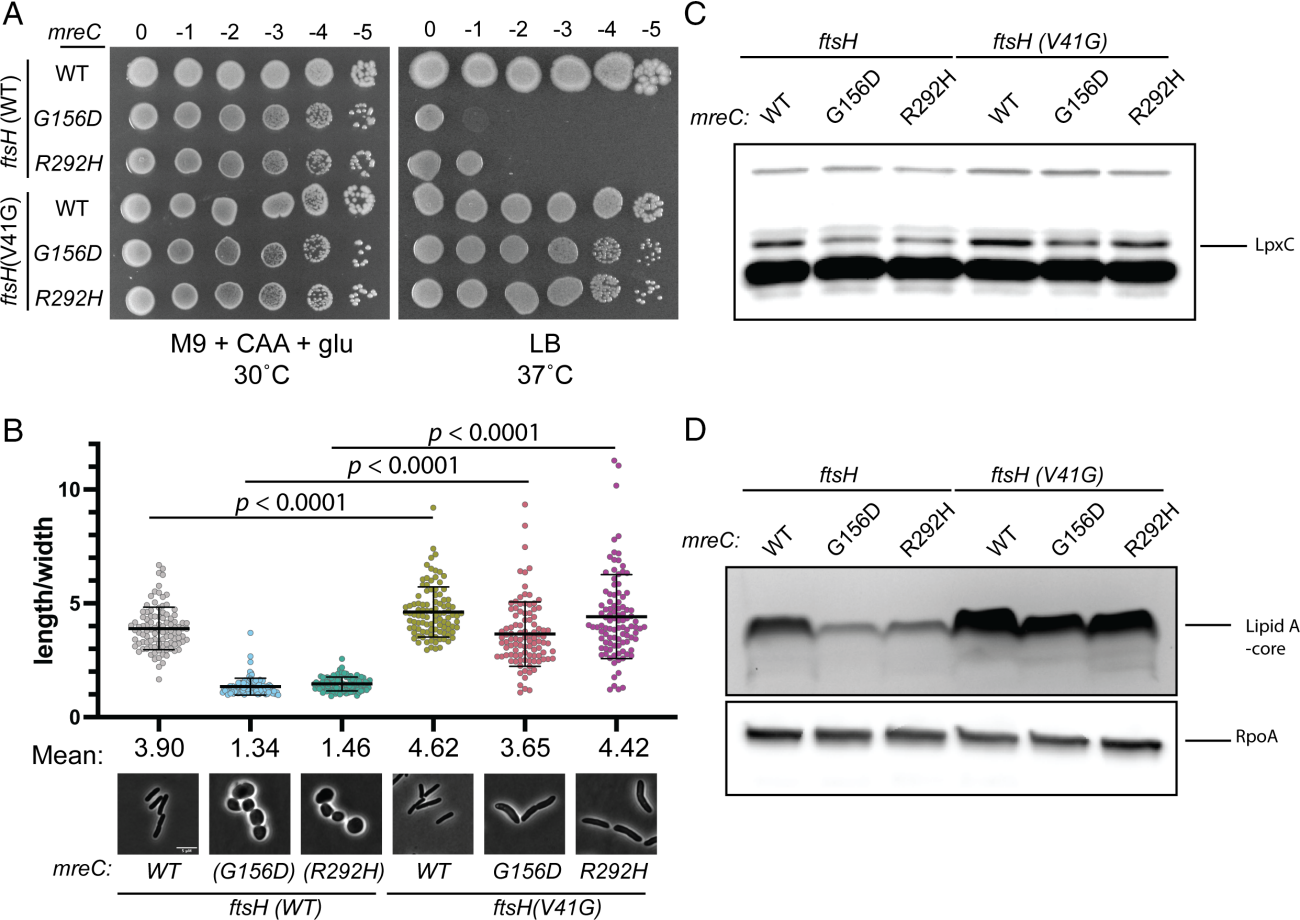
*FtsH(V41G)* increases LpxC and LPS levels in *mreC* hypomorphs. (*A*) Cultures of WT(EMF196), *mreC(G156D)* (EMF197), *mreC(R292H)* (PR109), *ftsH(V41G)* (EMF199), *mreC(G156D) ftsH(V41G)* (PR111), *mreC(R292H) ftsH(V41G)* (PR110) were incubated in M9 + CAA + glu at 30 °C for 24 h. Cultures were diluted and plated as in Fig. 1. (*B*) Cultures of the strains listed in (*A*) were diluted to OD_600_ = 0.05 in M9 + CAA + glu and incubated at 30 °C until OD_600_ = 0.2-0.3. Cultures were gently spun down and resuspended in LB to an OD_600_ = 0.025 and incubated at 37 °C until OD = 0.2-0.3. Cells were fixed and imaged (*Methods*). Aspect ratios were analyzed using the FIJI plugin MicrobeJ (39). (Scale bar, 5 µm.) n = 100 cells per group. Statistical significance was determined as in Fig. 1. (*C*) Cultures of the strains listed in (*A*) were grown as described in (*B*) and an immunoblot for LpxC was performed. (*D*) Cultures of the strains listed in (*A*) were grown as described in (*B*) and analyzed via silver stain for lipid A-core (*Top*). Samples were normalized to total protein and an immunoblot for RpoA was performed to serve as a loading control.

Both *ftsH* suppressors encoded protease variants with substitutions in the periplasmic loop of the protein (Fig. 1). One was found as a suppressor of *mreC(R292H)* and the other as a suppressor of *mreC(G156D)* (Fig. 1*B* and *SI Appendix*, Table S1). A mutation in *lapB* encoding a protein with a deletion of the last eleven C-terminal amino acids was also isolated as a suppressor of *mreC(G156D)* (Fig. 1*B* and *SI Appendix*, Table S1). Although growth rate and morphology were not restored to completely match those of wild-type cells, the suppressors supported full plating efficiency of their respective *mreC* mutant on LB (Fig. 1*B*) and switched their morphology from sphere-like to elongated rods (Fig. 1*C*). Suppression was not allele specific as the *ftsH(V41G)* mutation originally isolated as a suppressor of *mreC(G156D)* (*SI Appendix*, Table S1) also suppressed the growth and shape defects of *mreC(R292H)* (Fig. 2 *A* and *B*).

We chose to further characterize the mechanism of suppression by the *ftsH(V41G)* allele by determining its effect on the cellular concentration of LpxC (Fig. 2*C*) and LPS (Fig. 2*D*). In cells with wild-type FtsH, mutants encoding defective MreC variants had decreased levels of both LpxC (Fig. 2*C*) and LPS (Fig. 2*D* and *SI Appendix*, Fig. S1) compared to cells with MreC(WT). We also observed a similar dose-dependent decrease in LpxC levels following treatment with the MreB inhibitor A22, suggesting that a decrease in OM synthesis is not specific to the *mreC* mutants but instead is a more general response to perturbations to Rod complex activity (*SI Appendix*, Fig. S2). The *ftsH(V41G)* allele increased LpxC and LPS levels in cells with either hypomorphic allele of *mreC* (Fig. 2 *C* and *D* and *SI Appendix*, Fig. S1). This change resulted in an increase in LPS concentration to near normal in cells with the defective MreC variants (Fig. 2 *C* and *D* and *SI Appendix*, Fig. S1). RNAseq analysis showed that *lpxC* transcript levels remain unchanged in *mreC* mutants compared to WT, suggesting that the decrease in LpxC levels results from a change in posttranscriptional control (*SI Appendix*, Fig. S3). This finding is consistent with previous results showing that *lpxC* transcript levels remain the same under conditions when LpxC is stabilized, suggesting that LpxC production is not regulated at the transcriptional level (40). Although technical challenges resulting from the extremely low levels of LpxC in the *mreC* mutants prevented us from measuring its half-life in these strains, we think it is reasonable to conclude that the *ftsH(V41G)* allele is hypomorphic and likely leads to reduced LpxC turnover and a rise in LPS levels that compensates for the apparent defect in LPS synthesis of the *mreC* mutants.

**Fig. 3.**
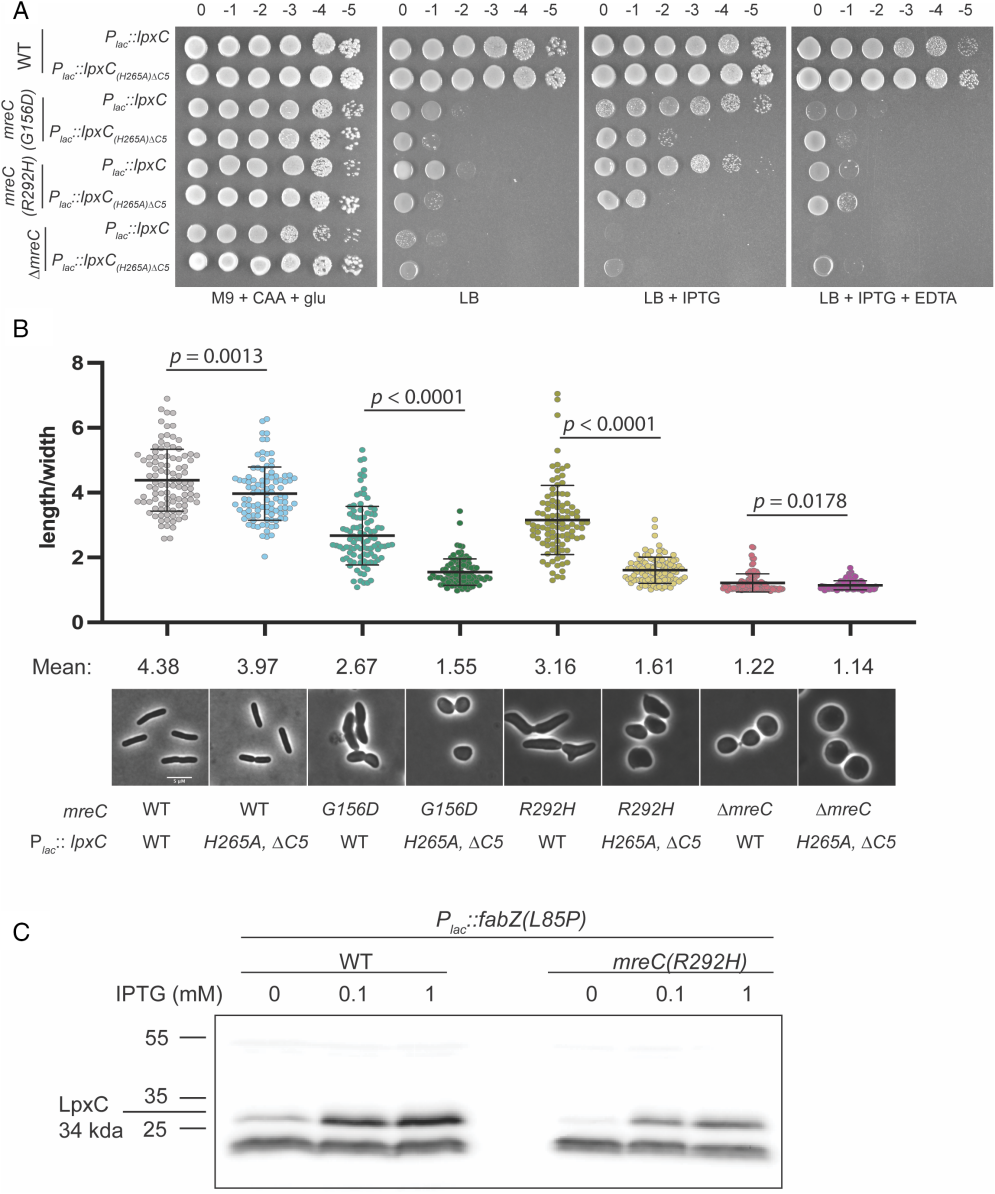
The overexpression of *lpxC* restores growth and partially restores shape to *mreC* hypomorphs. (*A*) WT (HC555), *mreC(G156D)* (PR30), *mreC(R292H)* (PR5), and *∆mreC* (EMF150) expressing WT *lpxC* (pPR111) or *lpxC(H265A)∆C5* (pPR115) from an IPTG-inducible plasmid were cultured for 24 h at 30 °C in M9 + CAA + glu. Cultures were diluted and plated on the indicated media as in Fig. 1. All plates contained CM. M9 plates were incubated at 30 °C for 40 h and LB plates were incubated at 30 °C for 24 h. (*B*) The strains listed in (*A*) were grown for 24 h at 30 °C in M9 + CAA + glu + CM. Cultures were diluted to OD_600_ = 0.025 in M9 + CAA + glu + CM + 50 µM IPTG and incubated at 30 °C until OD_600_ = 0.2-0.3. Cells were gently pelleted and resuspended in LB + CM + 50 µM IPTG and grown at 37 °C for 1 h 45 min. Cells were then fixed and imaged (*Methods*). Aspect ratios were analyzed using the FIJI plugin MicrobeJ (39). (Scale bar, 5 µm.) n = 100 cells per group. Statistical significance was determined as in Fig. 1. (*C*) Immunoblot for LpxC. Cell lysates of WT (HC555) and *mreC(R292H)* (PR5) cells harboring plasmids expressing *fabZ(L85P)* from an IPTG-inducible promoter (pEMF137) were cultured in M9 + CAA + glu + CM at 30 °C for 24 h. Cultures were then diluted to OD_600_ = 0.025 in M9 + CAA + glu + CM and grown at 30 °C until OD_600_ = 0.2-0.3. Cells were gently pelleted and resuspended in LB + CM ± IPTG as indicated and grown at 37 °C for 2 h and were subsequently harvested via centrifugation and processed for immunoblotting.

To determine whether an increase in LPS synthesis is sufficient to suppress the defective *mreC* alleles, we overexpressed *lpxC* in the mutants (Fig. 3). Overproduction of LpxC indeed promoted the growth of *mreC(R292H)* and *mreC(G156D)* mutants on LB and restored an elongated rod-like shape (Fig. 3). However, suppression was not as robust as that promoted by the *ftsH(V41G)* allele (Figs. 2 and 3), suggesting either that the levels of LPS upon LpxC overproduction were too high and caused mild toxicity or that changes in the turnover of FtsH substrates other than LpxC contribute to the suppressing activity of *ftsH(V41G).* Suppression was dependent on LpxC activity as the overproduction of a catalytically defective LpxC that lacks a degradation signal (designated as ∆C5) (29, 41, 42) failed to promote the elongated growth of cells producing the MreC variants (Fig. 3). Notably, overexpression of *lpxC* did not suppress an *mreC* deletion (Fig. 3), arguing that partial Rod complex activity in the *mreC(R292H)* and *mreC(G156D)* mutants is required to promote rod shape under suppressing conditions. Overall, our results suggest that the growth and shape defects of the *mreC(R292H)* and *mreC(G156D)* mutants is not just due to problems with PG biogenesis. Surprisingly, improper LPS synthesis and OM biogenesis also appear to be contributing factors. Notably, these findings explain previous reports of OM defects in mutants defective for the Rod system (11). Accordingly, we found that even under the permissive growth condition (minimal media, 30 °C), the *mreC* mutants are sensitive to a range of antibiotics indicating compromised OM barrier function (*SI Appendix*, Fig. S4).

### *mreC* Mutants Remain Capable of Sensing Perturbations to LPS Synthesis.

One explanation for the decrease in LPS production observed in the *mreC* mutants is that these cells are defective in modulating LpxC stability through the YejM/LapB/FtsH pathway in response to reduced LPS levels (30, 33–38). To test this possibility, we monitored LpxC levels following the overproduction of a hyperactive allele of *fabZ* (3-hydroxy-acyl-[acyl-carrier-protein] dehydratase) (29), an enzyme that functions early in the phospholipid synthesis pathway (43). Overproduction of this enzyme is expected to increase the flux of common precursors into the phospholipid synthesis pathway at the expense of LPS synthesis. Cells harboring the hyperactive *fabZ(L85P)* allele were previously reported to have increased levels of LpxC, presumably due to LpxC stabilization in order to restore balance between the two lipid biosynthesis pathways (29, 44). We found that *mreC(R292H)* cells overexpressing *fabZ(L85P)* had increased levels of LpxC compared to the uninduced controls and that the magnitude of the increase was comparable to that in WT cells upon induction of the hyperactive *fabZ* allele (Fig. 3*C*). We observed a similar result when we treated *mreC(R292H)* cells with the LpxC inhibitor CHIR-090 (45, 46), which was also previously shown to promote LpxC stabilization (47) (*SI Appendix*, Fig. S5). Thus, *mreC* mutant cells remain capable of sensing an acute reduction in LPS synthesis but fail to respond to and correct their chronic deficit in LpxC and LPS levels.

### OM Modifications Associated with Increased Stiffness Suppress Cell Shape Defects.

We reasoned that increasing LPS synthesis could suppress the shape defect of *mreC* mutants either by activating the Rod complex similar to previously characterized suppressors in *rodA* and *mrdA* encoding RodA-PBP2 (10) or by altering the structural properties of the OM. To test the former possibility, we measured the effect of the *ftsH(V41G)* allele on Rod complex activity in vivo using a radiolabeling assay. For this assay, a genetic background is used where PG synthesis by the divisome and the aPBPs can be inhibited by SulA production (48–50) and (2-sulfonatoethyl) methanethiosulfonate (MTSES) treatment (51), respectively. Rod complex activity can be further isolated by treatment with the PBP2-specific inhibitor mecillinam. This drug blocks the cross-linking activity of PBP2, but the glycosyltransferase activity of RodA remains active, leading to an accumulation of uncross-linked glycan chains. These uncross-linked glycans are known to be rapidly degraded by the lytic transglycosylase Slt (51). Thus, the accumulation of nascent PG turnover products during radiolabeling in the presence of mecillinam, MTSES, and SulA can be used as an indirect measure of Rod complex activity. Unlike the suppressing RodA and PBP2 variants characterized previously (10) that activate nascent PG turnover product accumulation, the *ftsH(V41G)* allele did not significantly alter Rod complex activity as assessed by the turnover assay (*SI Appendix*, Fig. S6). Furthermore, the activated PBP2(L61R) variant was found to increase the resistance of cells to the MreB inhibitor A22, another indication of its ability to activate the Rod complex. By contrast, overexpression of *lpxC* did not increase resistance to A22 (*SI Appendix*, Fig. S7). Taken together, these results suggest that hyperactivation of LPS synthesis does not suppress the shape and growth defects of *mreC* mutants by enhancing the PG synthesis activity of the Rod complex.

To investigate whether the mechanical stabilization of the OM is the underlying mechanism by which increased LPS synthesis restores shape to the *mreC* mutants, we sought alternative ways to alter OM stiffness. EDTA strips the OM of magnesium ions, disrupting the lateral interactions between adjacent LPS molecules (52), which has been shown to reduce cell envelope stiffness (3). The addition of EDTA reverses the growth benefit of *lpxC* overexpression in *mreC(R292H)* and *mreC(G156D)* mutants (Fig. 3*A*). We therefore conclude that LPS packing in the OM is required for the overexpression of *lpxC* to improve the growth of *mreC* hypormorphs (Fig. 3).

**Fig. 4.**
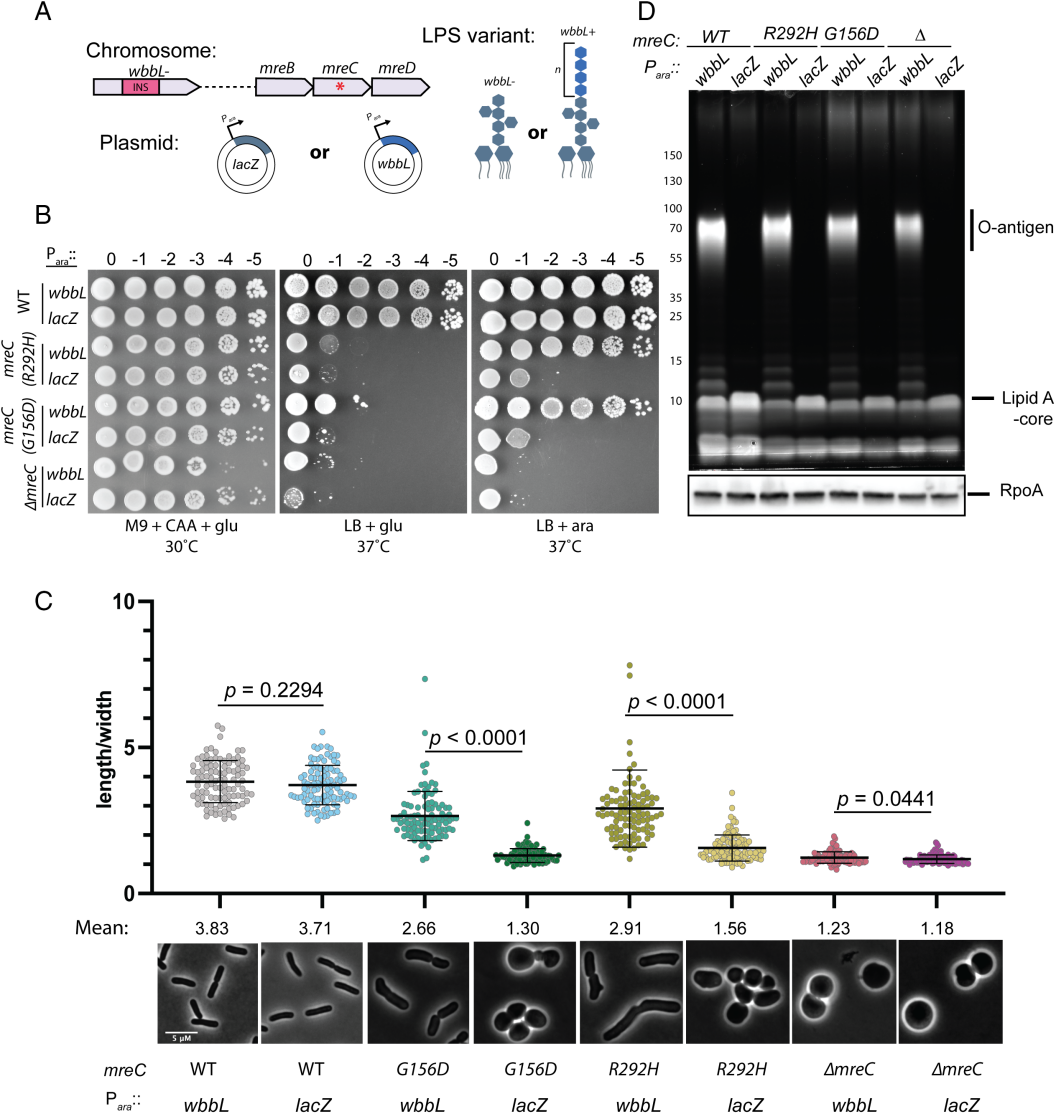
Synthesis of O-antigen-modified LPS suppresses the growth and shape defects of *mreC* hypomorphs. (*A*) Schematic of strains. The *wbbL* gene in *E. coli* K-12 is disrupted by an insertion element, preventing the synthesis of O-antigen. *wbbL* is expressed *in trans* from an arabinose (ara)-inducible promoter, restoring O-antigen synthesis. *lacZ* is expressed as a control. (*B*) WT (HC555), *mreC(R292H)* (PR5), *mreC(G156D)* (PR30), and *∆mreC* (EMF150) expressing *wbbL* (pEMF130) or *lacZ* (pEMF134) from an arabinose-inducible promoter were incubated for 24 h in M9 + CAA + glu + tet at 30 °C. Cultures were diluted and plated on the indicated media as in Fig. 1. (*C*) The strains listed in (*A*) were grown for 24 h in M9 + CAA + glu + tet at 30 °C and diluted to OD_600_ = 0.05 in M9 +CAA + ara + tet for 3 h at 30 °C. After 3 h, the cultures were gently pelleted and resuspended in LB + tet + ara. Cells were grown for 2 h at 37 °C. Cells were then fixed and imaged (*Methods*). Aspect ratios were analyzed using the FIJI plugin MicrobeJ (39). (Scale bar, 5 µm.) n= 100 cells per group. Statistical significance was determined as in Fig. 1. (*D*) Proemerald Q stain of LPS. The strains listed in (*A*) were grown as described in (*B*). Cell lysates were prepared and LPS was analyzed via promerald Q straining. Note: in our experience, the proemeraldQ method of detecting LPS allowed for more consistent visualization of high molecular weight O-antigen modified species compared the silver stain method used in Fig. 2.

We next investigated the effect of increasing OM stiffness by reintroducing O-antigen in the *mreC* mutants. LPS is composed of three covalently attached units (53). The base glycolipid is called Lipid A. It is modified by a core oligosaccharide that is conserved among Gram-negative organisms. The core is further modified by longer polysaccharide chains called O-antigens, the composition of which varies between species. Laboratory strains of *E. coli* K-12 do not synthesize O-antigen due to an insertion element in *wbbL* (54), a gene required for producing LPS modified by the O-16 O-antigen serotype (52). It was previously reported that restoring O-antigen to the OM dramatically increases its stiffness (3). We therefore asked if reintroducing wild-type *wbbL* to the *mreC* mutants on an arabinose-inducible plasmid could suppress their growth and shape phenotypes like the overexpression of *lpxC* (Fig. 4*A*). Expression of *wbbL* but not a *lacZ* control promoted growth of the *mreC* hypomorphs under the nonpermissive condition (LB, 37 °C) and restored their growth as elongated rods (Fig. 4 *B* and *C*). Importantly, we did not observe an increase LpxC levels in WT or *mreC(R292H)* cells expressing *wbbL* compared to the *lacZ* control (*SI Appendix*, Fig. S8*A*), indicating that *wbbL* is not acting by directly increasing LPS synthesis but rather is improving the structure of the envelope by increasing lateral interactions between adjacent LPS molecules in the OM. We also observed that *wbbL* can partially rescue the shape defects of *mreC(R292H)* cells when expressed at the native locus, although not to the same extent as expression from a multicopy plasmid (*SI Appendix*, Fig. S8 *B* and *C*). This suppression phenotype is further improved by overexpressing *lpxC*, suggesting both methods of increasing OM stiffness have an additive effect on cell shape improvement. As we observed with cells overexpressing *lpxC*, overexpressing *wbbL* did not improve the shape or growth defects of *∆mreC* cells even though they synthesized comparable levels of O-antigen-LPS as the other strains (Fig. 4*D*). Restoring O-antigen synthesis also did not restore shape to cells deleted for *rodZ* (*SI Appendix*, Fig. S9). Therefore, an intact Rod complex is required to mediate the growth and shape changes in mutant cells with a restored O-antigen. From these results, we infer that OM stiffening is the likely mechanism by which changes in LPS synthesis or modification restores rod shape to cells with a poorly functioning Rod complex.

### OM Stiffness and the Directional Motion of MreB Filaments.

**Fig. 5.**
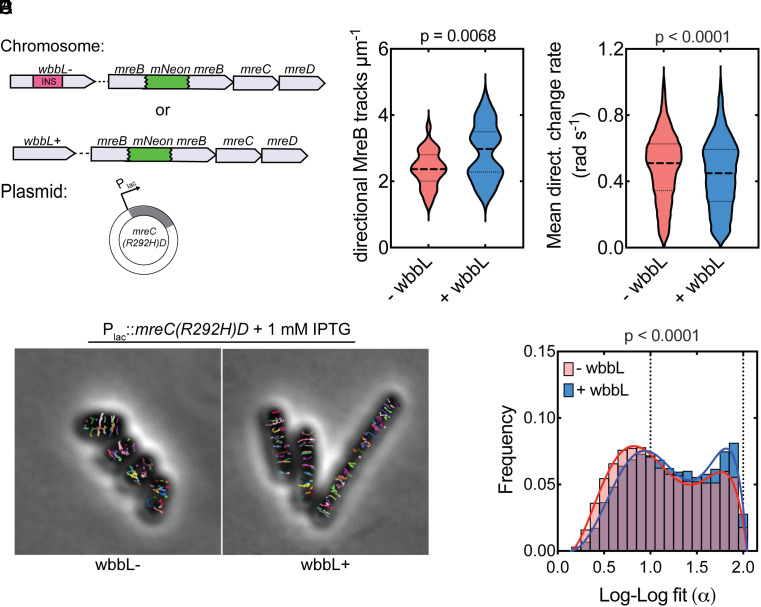
MreB dynamics upon Rod complex inactivation by *mreC(R292H)* in cells with or without O-antigen. (*A*) Schematic of strains. *^SW^mreB-mNeon* cells harbor either *wbbL*(INS) (AV007) or *wbbL*+ (EMF210) at the native chromosomal locus, resulting in cells without or with O-antigen-modified LPS, respectively. *mreC(R292H)(D)* is expressed in trans from an IPTG-inducible promoter (pMS9). (*B*) *wbbL(INS)* (AV007) or *wbbL+* (EMF210) cells expressing *mreC(R292H)D* (pMS9). Individual traces of MreB tracks were mapped using the TrackMate feature of FIJI (56, 57). Each track is indicated in a different color. (*C*) Violin plot of the number of directional MreB tracks per cell area in cells with (EMF210) and without O-antigen (AV007) expressing *mreC(R292H)D* (pMS9). [n = 30 cells (AV007), n = 31 cells (EMF210)]. Statistical significance determined by an unpaired *t* test with Welch’s correction. (*D*) Violin plot of the mean directional change rate of MreB tracks in *wbbL* (−) and *wbbL* (+) cells [n = 10,214 tracks (AV007), n = 9,162 tracks (EMF210)]. Statistical significance determined by the Mann–Whitney test. (*E*) Histogram of the log-log fit (α) values of Individual MreB traces in cells with (EMF210) and without O-antigen (AV007) expressing *mreC(R292H)D* (pMS9). [n = 18,618 tracks (AV007), n = 15,070 tracks (EMF210)]. Statistical significance determined by the Mann–Whitney test.

MreB polymers align along the greatest principal curvature of the cell and are thought to orient the insertion of new PG by the Rod complex perpendicular to the long cell axis via a rudder-like mechanism (55). MreB polymers thus promote growth in a rod shape, but they also require rod shape for their proper alignment. Rod shape is therefore thought to be a self-reinforcing property (21). We reasoned that this rod-shape feedback loop is impaired in the *mreC* mutants because the reduced activity of the Rod complex fails to build an envelope robust enough to maintain the beginnings of a cylindrical extrusion that can be elongated into a rod via oriented MreB motion. However, strengthening of the OM in the suppressors may overcome this problem by stabilizing the envelope, allowing a partially functional machine to promote the self-enhancing shape determination process. To test this hypothesis, we wanted to track the motion of a functional MreB-mNeon sandwich fusion (^SW^MreB-mNeon) (7) in *mreC* hypomorphic cells with and without shape-restoring suppressor mutations. Unfortunately, we were unable to construct strains encoding both the *mreC* hypomorphic alleles and the *^SW^mreB-mNeon* fusion at the native locus because the combination was toxic. Instead, we produced ^SW^MreB-mNeon from the native *mreB* locus that also contained *mreC(WT)* and overexpressed the dominant-negative *mreC(R292H)* allele from a plasmid in cells with or without O-antigen (Fig. 5*A*). Overexpression of *mreC(R292H)* caused cells lacking O-antigen to form sphere-like cells, but the shape change was not as dramatic as that observed for cells harboring *mreC(R292H)* as the sole copy of the gene at the native locus. As expected, rod shape was maintained in O-antigen positive cells overexpressing *mreC(R292H).* In addition to the differences in shape, the presence of O-antigen also impacted MreB dynamics. Compared to the rod-shaped O-antigen positive cells, cells lacking O-antigen showed a reduction in the number of directionally moving particles and those particles that were moving did not appear to have as consistent of an orientation (Fig. 5 *B*, *C*, and *E* and Movie S1). Particles in the O-antigen positive cells were also less likely to change direction during imaging than those in the cells lacking O-antigen (Fig. 5*D*). These results argue that the OM contributes to shape determination by providing sufficient envelope stability for MreB-directed PG synthesis to be properly oriented and self-reinforcing.

## Discussion

The OM and PG layers of the Gram-negative envelope share numerous connections. Their building blocks are synthesized from common precursors (58–60), and the layers are physically linked by PG-binding proteins anchored in the OM (61–63). Additionally, the insertion of beta-barrel proteins in the OM appears to be spatially coordinated with the insertion of new PG material into the mature cell wall matrix (64). Despite these connections, it has only recently been appreciated that the OM plays a role in the mechanical stability of the Gram-negative envelope that rivals that of the cell wall (3, 65–67). Here, we provide evidence that rather than just stiffening the envelope, the OM also plays a critical role in rod shape determination. Additionally, our genetic analysis uncovered an unexpected connection between LPS synthesis and the activity of the Rod complex that elongates the PG matrix, revealing yet another link between the two outermost layers of Gram-negative cells.

A morphogenic role for the OM is inferred from the ability of elevated LPS synthesis or O-antigen modification to restore rod-like shape to cells with a partially defective Rod complex. The shape mutants showed a reduced level of LPS and the LPS synthesis enzyme LpxC (Fig. 2). The stiffness of the OM is thought to be mediated by the lateral packing of LPS molecules bridged by Mg^2+^ ions (3). Thus, the OM of the shape defective cells with reduced LPS likely has suboptimal LPS packing and reduced stiffness. Increasing LPS synthesis in these cells by stabilizing LpxC or overproducing it is expected to increase the LPS concentration in the OM of these cells, enhancing lateral interactions between LPS molecules to at least partially restore OM mechanical stability (Fig. 3*A*). Similarly, the addition of O-antigen is likely to stiffen the membrane despite suboptimal LPS levels because the extended glycan chains facilitate long-distance LPS–LPS interactions.

**Fig. 6.**
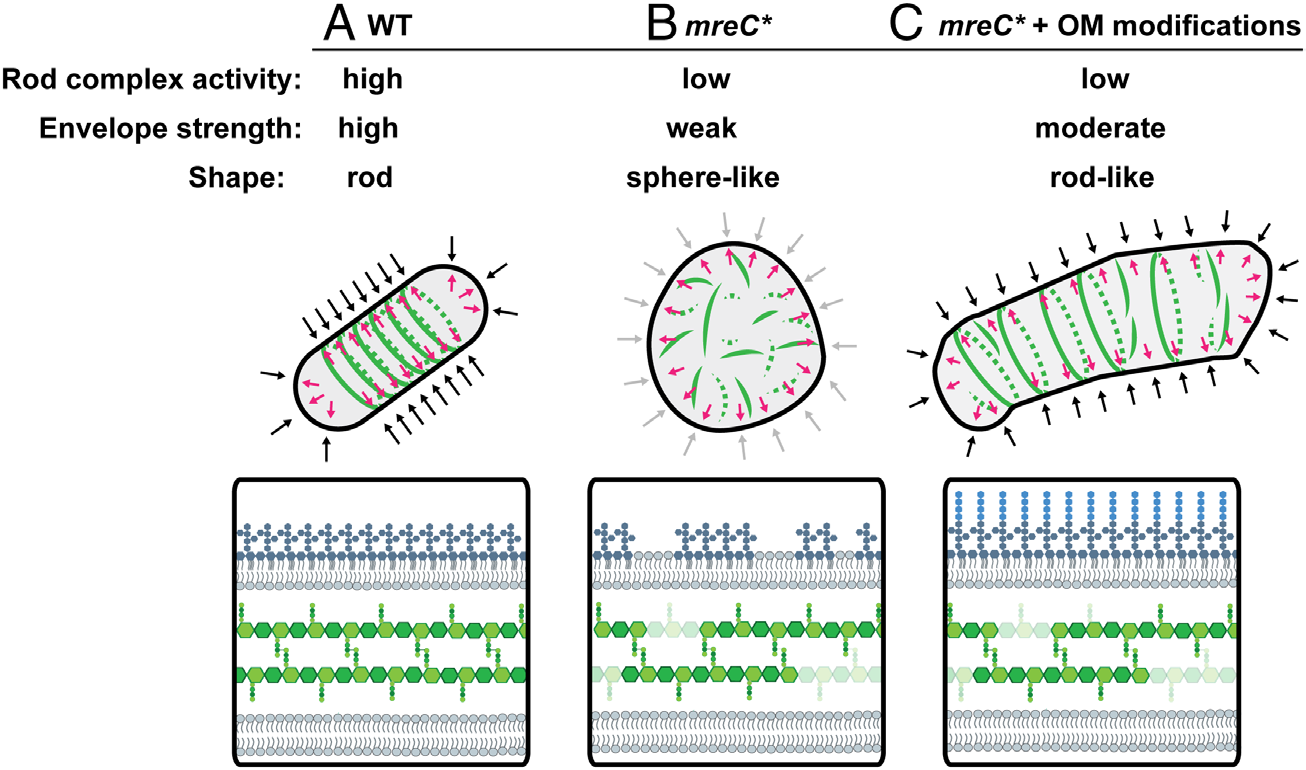
Interventions that strengthen the OM restore shape to Rod complex hypomorphs. (*A*) In wild-type cells, the internal turgor pressure of the cell is countered by the combined mechanical strength of the cell wall and the OM. The Rod complex is fully functional and is orientated by MreB, which aligns along the greatest principle curvature to ensure synthesis perpendicular to the long axis of the cell. (*B*) In hypomorphic *mreC* mutants (mreC*), the Rod complex is not able to synthesize sufficient PG or LPS, weakening the envelope and leading to loss of rod shape. The cells no longer form a clearly defined long axis, causing MreB filaments to misalign. The reduced Rod complex activity in these mutants is therefore not properly oriented. (*C*) When the mechanical strength of the OM is increased, the cell envelope is sufficiently able to resist the internal turgor pressure of the cell to allow for the initiation and propagation of a rod shape by allowing MreB and limited PG synthesis by the Rod complex to properly orient.

How does OM stiffening rescue the Rod complex defect? We propose that it does so by promoting the oriented-synthesis feedback via which the Rod complex generates rod shape (21) (Fig. 6). A critical feature of this model of shape determination is that rod shape is self-reinforcing due to the curvature preference of MreB filaments that orients them perpendicular to the long cell axis to guide PG synthesis by the Rod complex (55). If the cell wall made by the machinery is not stiff enough to hold the beginnings of a cylindrical shape in the face of turgor pressure, as is likely the case in the *mreC* mutants, then the feedback loop that elongates the cylinder to generate rod shape cannot be initiated (Fig. 6). This is reminiscent of a similar phenomenon in Gram-positive bacteria with defects in wall teichoic acid (WTA) synthesis (55). Much like LPS, these anionic cell wall polymers have been proposed to stiffen the envelope at least in part through lateral interactions mediated by bridging Mg^2+^ ions (21). Accordingly, mutants with reduced levels of WTA synthesis can be converted from rods to spheres by removing Mg^2+^ from the medium (55). Moreover, cell shape can be restored to *B. subtills* mutants with a partially defective Rod complex by the addition of excess Mg^2+^(68, 69). Although it remains to be determined whether the mechanism behind shape restoration in this context is based on envelope rigidification or potential effects on the activity of PG cleaving enzymes, the parallels suggest the attractive possibility that the LPS of Gram-negative bacteria and WTAs of Gram-positive organisms may function similarly to promote cell shape by providing sufficient envelope rigidity to enable the self-reinforcing orientation of PG synthesis by the Rod complex.

Given its relevance to antibiotic resistance, the most well-studied role of the OM is as a permeability barrier preventing the entry of bulky and/or hydrophobic drugs. Mutants defective for the Rod complex have been known to have a defective OM permeability barrier for many years (11, 70), but the cause of their increased permeability to antibiotics has been unclear. Our results indicate that the problem is likely caused by a reduction in LPS synthesis in the spherical cells. Whether this reflects a direct or indirect connection between Rod complex activity and the LPS synthesis and/or transport systems is not known. However, the *mreC* mutants we studied are still capable of responding to reductions in the flux through the LPS synthesis pathway by stabilizing LpxC (Fig. 3*C* and *SI Appendix*, Fig. S5). Thus, the defect does not appear to be at the level of the YejM-LapB-FtsH system that monitors the steady-state level of LPS in the outer leaflet of the IM (30, 33–38). One possible model is that LPS synthesis is down-regulated in Rod complex mutants in order to direct common precursors toward PG synthesis in an attempt to restore cell wall integrity. In this case, the cell may be triaging PG synthesis at the expense of the OM. Our results indicate that reduced LPS synthesis in this case is not due to differential transcriptional regulation of *lpxC*. Further study will be needed to determine whether the regulation works through the YejM-LapB-FtsH pathway or through a different mechanism and to understand if and how the status of the cell wall is sensed as part of the regulatory systems governing OM biogenesis. Notably, several studies have also recently made connections between PG and OM synthesis in *Pseudomonas aeruginosa* (71), *Acinetobacter baumannii* (72), and *Vibrio cholera* (73). Future investigation of these and other PG-OM connections promises to reveal new ways to compromise the permeability barrier of diverse Gram-negative bacteria to sensitize them to antibiotics.

## Methods

### Bacterial Strains and Growth Conditions.

The strains generated and used in this study are derivatives of MG1655 and cultured in LB (1% tryptone, 0.5% yeast extract, 0.5% NaCl) or minimal (M9) medium (74). Minimal medium was supplemented with 0.2% Casamino Acids (CAA) and 0.2% glucose (glu) or arabinose (ara) where indicated (see figure legends). Rod complex mutants and controls were maintained on M9 + CAA + glu at 30 °C unless otherwise indicated. Strains harboring plasmids were grown in the presence of antibiotics at the following concentrations (unless indicated differently in the figure legends): 25 µg/mL chloramphenicol (CM), 25 µg/mL kanamycin (Kan), and 10 µ/mL tetracycline (Tet). All strains, plasmids, and primers used in this study are listed in *SI Appendix*, Tables S2, S3, and S4, respectively. For details, please see *SI Appendix*, *Supporting text*.

### Suppressor Analysis.

Suppressors were isolated and analyzed as described previously (10).

### Western Blots.

Cells were pelleted via centrifugation and resuspended in water and 2× Laemmli sample buffer (100 mM Tris-HCl, pH 6.8; 2% SDS; 0.1% bromophenol blue; 20% glycerol) at a 1:1 ratio to a final OD_600_ of 20, boiled for 10 min, and stored at −80 °C. Samples were thawed and sonicated for 1 min twice using a Qsonica tip sonicator with an amplification of 25%. Sample concentration was determined using the Noninterfering (NI) Protein Assay [with bovine serum albumin (BSA) protein standard] (G Biosciences catalog no. 786-005). Samples were run on a 15% polyacrylamide gel (LpxC western blots) or 4 to 20% Mini-PROTEAN gels (BioRad cat# 4568095) and transferred to a polyvinylidene difluoride membrane. The membrane was rinsed in phosphate-buffered saline containing 0.1% Tween (PBS-T) [10% 10× PBS-T buffer, pH 7.4 (Sigma-Aldrich)] and blocked in 5% milk in PBS-T for 1.5 h. The membrane was incubated in 1% milk-PBS-T containing rabbit anti-LpxC antibody (a generous gift from the Doerrler lab) or mouse anti-RpoA (anti- *E. coli* RNA polymerase alpha from Biolegend, cat# 663104) diluted 1:10,000. The membranes were incubated at 4 °C O/N rocking and then washed 4× with PBS-T at room temperature (1× quickly followed by 3× for 10 min). For LpxC blots, the membrane was incubated in 0.2% milk dissolved in PBS-T with [HRP]-conjugated anti-rabbit IgG (1:40,000 dilution, Rockland cat# 18–8816-33). For RpoA western blots, membranes were incubated with anti-mouse IgG HRP at a dilution of 1:3,000 (Thermo Fisher Scientific catalog no. 34577). Membranes were incubated with secondary antibody for 2 h and then washed 5× with PBS-T (1× quickly followed by 4× for 10 min per wash). Membranes were developed using the SuperSignal West Pico Plus chemiluminescent substrate (Thermo Fisher Scientific catalog no. 34577) and imaged using the c600 Azure Biosystems platform.

### Detecting LPS Using Silver Stain.

Cultures were prepared as described in figure legends. For Fig. 2 and *SI Appendix*, Fig. S1, strains listed in the figure legend were cultured for 24 h at 30 °C in M9 + CAA + glu. Cultures were then diluted to OD_600_ = 0.05 and grown at 30 ˚C until OD = 0.2-0.3. Cells were gently pelleted and resuspended in LB (OD_600_ = 0.025) and grown at 37 °C until OD_600_ = 0.2-0.3. Cells were pelleted and resuspended in 1× LDS sample buffer (Invitrogen NP0008) + 4% -mercaptoethanol) to a final OD_600_ of 20. Pellets were boiled for 10 min and stored at −80 °C. The protein concentration of the samples was measured using the Noninterfering (NI) Protein Assay (with BSA protein standard) (G Biosciences catalog no. 786-005). RpoA western blots were carried out as described above. For the LPS silver stain, 50 µL of sample was incubated with 1.25 µL of proteinase K (NEB P8107S) for 1 h at 55 °C and then 95 °C for 10 min. Also, 20 µg (volume equivalent) was resolved on a 4 to 12% Criterion XT Bis-Tris gel (Bio-Rad 3450124) at 100V for 2 h. LPS detection via silver stain was performed as described previously (75). First, the gel was fixed overnight in a solution of 200 mL of 40% ethanol and 5% acetic acid. Periodic acid was added to the fixative solution (final concentration of 0.7%). Following a 5 min incubation at room temperature, the gel was washed with 200 mL ultrapure H_2_0 (2× for 30 min, 1× for 1 h). The gel was then incubated with 150 mL of staining solution (0.018 N NaOH, 0.4% NH_4_OH, and 0.667% Silver Nitrate) for 10 min. The gel was then washed 3× for 15 min in 200 mL ultrapure H_2_0 and developed in developer solution (0.26 mM Citric Acid pH 3.0, 0.014% formaldehyde). The reaction was stopped by removing the developer and replacing it with 100 mL of 0.5% acetic acid. The gel was imaged using the Bio-Rad ChemiDocTM MP Imaging System.

### Detecting LPS Using the Pro-Q Emerald 300 LPS Gel Stain Kit.

WT (HC555), *mreC(R292H)* (PR5), *mreC(G156D)* (PR30), and *∆mreC* (EMF150) expressing *wbbL* or *lacZ* from an arabinose-inducible promoter were incubated for 24 h in M9 + CAA + glu + tet at 30 °C and diluted to OD_600_ = 0.05 in M9 +CAA + ara + tet for 3 h at 30 °C. After 3 h, the cultures were gently pelleted and resuspended in LB + ara + tet. Cells were grown for an additional 2 h at 37 °C. Cells were pelleted and resuspended in 1× LDS sample buffer (Invitrogen NP0008) + 4% 2-mercaptoethanol) to a final OD_600_ of 20, boiled for 10 min, and stored at −80 °C. The protein concentration of the samples was measured using the Noninterfering (NI) Protein Assay (with BSA protein standard) (G Biosciences catalog no. 786-005). RpoA western blots were carried out as described above. For the LPS proemeraldQ stain, 50 µL of sample was incubated with 1.25 µL of proteinase K (NEB P8107S) for 1 h at 55 °C then 95 °C for 10 min. A normalized volume equivalent to 20 µg total protein in the predigested sample was resolved on a 4 to 12% Criterion XT Bis-Tris gel (Bio-Rad 3450124) at 100V for 2 h. The Proemerald Q stain was performed following the manufacturer’s instructions (Pro-Q Emerald 300 LPS gel stain kit-Molecular Probes P20495). The gel was imaged using the Bio-Rad ChemiDocTM MP Imaging System.

### Phase Contrast Microscopy.

Phase contrast micrographs in Figs. 1, 2, 3, and 4 and *SI Appendix*, Figs. S2, S8, and S9 were all taken using cells fixed in 2.6% in formaldehyde and 0.04% glutaraldehyde. After adding the fixative, cells were incubated at room temperature for 1 h and stored at 4 °C for a maximum of 3 d. To image, cells were immobilized on agarose pads (2%) on 1 mm glass slides (1.5 coverslips). Micrographs in Fig. 1 were taken using a Nikon TE2000 inverted microscope using a 1.4 NA Plan Apo Ph3 objective and Nikon Elements Acquisition Software AR 3.2. Micrographs in Fig. 2 were taken with a Nikon Ti Inverted Microscope using a 1.4 NA Plan Apo 100× Ph3 DM objective and with Nikon Elements 4.30 Acquisition Software. Micrographs in Figs. 3 and 4 and *SI Appendix*, Figs. S2, S8, and S9 were taken with a Nikon Ti2-E inverted microscope using a 1.45 NA Plan Apo 100× Ph3 DM objective lens and Nikon Elements 5.2 Acquisition Software. Micrographs were processed using rolling ball transformation (radius = 35 pixels) in FIJI (76) prior to length and width quantification using the microbeJ plugin (39). The aspect ratio was calculated by dividing the length measurements by the width measurements. The data were plotted in GraphPad Prism and statistical analysis of aspect ratio done in GraphPad Prism using a parametric unpaired *t* test assuming gaussian distribution but not equal SD (Welch’s correction). Images were cropped in FIJI (76).

#### 3H-mDAP physiological radiolabeling.

PG turnover was determined as described previously (7, 10, 51). Data were plotted on GraphPad Prism.

### MreB Dynamics.

*wbbL(INS)* (AV007) or *wbbL+* (EMF210) cells expressing *mreC(R292H)D* (pMS9) were back diluted from overnight cultures (1:200) and grown in LB + 1 mM IPTG and incubated at 37 °C until OD_600_ = ~0.4. Cells were then back diluted a second time to OD_600_ = 0.05 in LB + 1 mM IPTG and incubated at 37 °C until OD_600_ = ~0.4. # 1.5 high precision coverslips (Marienfeld) were added to a hydrochloric acid and ethanol and cleaned. Cells were placed onto a 2% (w/v) agarose pad in LB + 1 mM IPTG and imaged at RT on a Nikon Ti inverted microscope equipped with Nikon TIRF Lun-f laser illumination, a Plan Apo 100×, 1.45 NA Ph3 objective lens. Images were recorded using an Andor Zyla 4.2 Plus sCMOS camera and Nikon Elements 4.30 acquisition software. Three-minute timelapse series with an acquisition frame rate of 3s were recorded to capture MreB dynamics and overlayed over a single-frame phase contrast reference image using Fiji (76). Particle tracking was performed as described in Navarro et al. (77). Briefly, MreB tracks were detected in TrackMate v6.0.1 (56) using a LoG detector (0.3-µm radius) and the Kalman filter. To analyze the nature of the displacement of each track, the mean square displacement ( MSD ) was calculated using the MATLAB class msdanalyzer (78). Slopes ( α ) of the individual MSD curves were extracted using the Log-log fit of the MSD and the delay time τ . As the maximum delay time 75% of the track length was used. Only tracks which persisted for longer than 4 timepoints (12 s) and with a $R^{2}\;\mathrm{for}\;\log\;\left[MSD\right]\;$ versus $log\;\left[t\right]$ above 0.95 were included in the analysis. MreB filaments engaged in active cell wall synthesis are displaced by the action of the enzymatic activities of RodA and PBP2 (2, 7, 17–20, 22, 79) and thus its MSD curves display slopes of α ≈ 2 indicative of a transported particle motion above the rate of Brownian diffusion ( α ≈ 1) or confined motion ( α > 1). The mean directional change rate was derived from TrackMate and is defined as a measure of the angle between two succeeding links, averaged over all the links of a track, and is reported in radians.

### RNAseq Analysis.

Samples were prepared as described in the figure legend (*SI Appendix*, Fig. S3) and sent to SeqCenter (https://www.seqcenter.com/). RNA extraction, sequencing, and analysis were performed by SeqCenter following standard protocols. Briefly, samples were treated with DNAse (Invitrogen, RNAse free) and libraries were prepared using Illumina’s Stranded Total RNA Prep Ligation with the Ribo-Zero Plus kit and 10 bp unique dual indices. Sequencing was performed on a NovaSeq 6000, generating paired-end 151-bp reads. Demultiplexing, quality control, and adapter trimming was performed with bcl-convert (v4.1.5). *lpxC* transcripts were normalized to WT (HC555) and plotted using GraphPad Prism.

## Supplementary Material

Appendix 01 (PDF)

Movie S1. **Time lapse of MreB in *wbbL(INS)* (AV007) and *wbbL (+)* (EMF210) cells expressing *mreC(R292H)D* (pMS9) described in Fig. 5.** Three-minute timelapse series with an acquisition frame rate of 3s were recorded to capture MreB dynamics. TOP: ^*SW*^*mreB-mNeon* overlayed over a single-frame phase contrast reference image. BOTTOM: Examples of MreB tracks identified using TrackMate (6, 7). Scale Bar = 2 μM.

## Data Availability

The data that support the findings of this study as well as the associated protocols are all presented in the paper. Bacterial strains and other reagents generated during the course of this study are available from the corresponding author upon reasonable request.
