# Supplementary material for: A role for the Gram-negative outer membrane in bacterial shape determination
Source: Proc Natl Acad Sci U S A. 2023 Aug 22;120(35):e2301987120. doi: 10.1073/pnas.2301987120 (PMC10469335; doi:10.1073/pnas.2301987120)
Supplement: Supplementary file 1 — Appendix 01 (PDF) [file pnas.2301987120.sapp.pdf]

Supporting Information for:

A role for the Gram-negative outer membrane in bacterial shape determination

Authors: Elayne M. Fivenson[1], Patricia D.A. Rohs[1], Andrea Vettiger[1], Marios F. Sardis[1], Grasiela Torres[1], Alison Forchoh[1], and Thomas G. Bernhardt[1,2*]

Affiliations:
[1]Department of Microbiology, Blavatnik Institute, Harvard Medical School, Boston, MA 02115.
[2]Howard Hughes Medical Institute, Chevy Chase, MD, United States.

***To whom correspondence should be addressed**.
Thomas G. Bernhardt, Ph.D.
Harvard Medical School
Department of Microbiology
Boston, Massachusetts 02115
e-mail: thomas_bernhardt@hms.harvard.edu

This PDF file includes:

Supporting Text
Figures S1 to S9
Legend for Movie S1
Tables S1 to S4
SI references

Other supporting materials for this manuscript include the following:

Movie S1

Supporting text

Molecular biology

The polymerase chain reaction (PCR) was carried out using Q5 High fidelity polymerase (New England Biolabs) or GoTaq green master mix (Promega) following manufacturer's instructions. PCR products were purified using the PCR clean up kit from Qiagen or CWBiosciences. Plasmids were isolated using the Miniprep Kit from Qiagen or the plasmid purification kit from CWBiosciences.

Strain construction details

PR82
See supplementary table 1 and Methods

PR86
See supplementary table 1 and Methods

PR88
See supplementary table 1 and Methods

EMF196
The *yrdE-kan* allele from strain HC555 was transduced into strain PR103 by P1-mediated transduction. Transductants were selected on LB + kan25 and confirmed for the yrdE-kan allele via PCR.

PR103
The *leuU-cat-yhbX* allele from strain PR90 was transduced into MG1655 by P1-mediated transduction. Transductants were selected on LB + CM25.

PR90
A chloramphenicol resistance cassette was introduced to strain TB10 between *leuU* and *yhbX* (linked to *ftsH*) by lambda red recombineering. A PCR product amplified from pKD3 was generated using Primers leuU-yhbX_P2_F (ACCTTGAAACGATGGTGCCGGTACGCCTTAGTTATAAATTCATATGAATATCCTCCTTAG) and leuU-yhbX_P1_R (TTGACACAATAAAGTGCCAATTATGTCAGTAGAAGGGAAAGTGTAGGCTGGAGCTGCTTC) and electroporated into strain TB10 following the protocol for strain DY329 described previously(1).

EMF197
The *yrdE-kan* marker linked to the *mreC(G156D)* allele from strain PR30/pTB63 was transduced into strain PR103 via P1-mediated transduction. Transductants were selected on M9 + CAA + glu + kan25. The *mreC(G156D)* allele was confirmed via sequencing.

EMF199
The *yrdE-kan* allele from strain HC555 was transduced into strain PR104 by P1-mediated transduction. Transductants were selected on LB + kan25 and confirmed for the *yrdE-kan* allele via PCR.

PR104
The *leuU-cat-yhbX* allele linked to *ftsH(V41G)* from strain PR96 was transduced into MG1655 via P1-mediated transduction. Transductants were selected on LB + CM25 and confirmed for the *ftsH(V41G)* allele via sequencing.

PR96
A chloramphenicol resistance cassette was introduced to strain PR88 between *leuU* and *yhbX* (linked to *ftsH*) by lambda red recombineering with pKD46 plasmid following the protocol described previously (2) . A PCR product amplified from pKD3 using primers leuU-yhbX_P2_F (ACCTTGAAACGATGGTGCCGGTACGCCTTAGTTATAAATTCATATGAATATCCTCCTTAG) and leuU-yhbX_P1_R (TTGACACAATAAAGTGCCAATTATGTCAGTAGAAGGGAAAGTGTAGGCTGGAGCTGCTTC) and electroporated into strain PR88/pKD46.

PR109
The *yrdE-kan* marker linked to *mreC(R292H)* from strain PR5/pTB63 was transduced into strain PR103 by P1-mediated transduction. Transductants were select on M9 + CAA + glu + kanamycin.

PR111
The *yrdE-kan* marker linked to the *mreC(G156D*) allele from strain PR30/pTB63 was transduced into strain PR104 via P1-mediated transduction. Transductants were select on M9 + CAA + glu + kanamycin. The *mreC(G156D*) allele was confirmed via sequencing

PR110
The *yrdE-kan* marker linked to *mreC(R292H)* from strain PR5/pTB63 was transduced into strain PR104 by P1-mediated transduction. Transductants were select on M9 + CAA + glu + kanamycin. The *mreC(R292H*) allele was confirmed via sequencing.

EMF150
The Δ*mreC::kan* allele from strain MT4/pTB63 was transduced into MG1655 via P1-mediated transduction. Transductants were select on M9 + CAA + glu + kanamycin.

AV007
The chloramphenicol resistance cassette in strain JAB593 was cured using pcp20 as described previously (3).

EMF210
The *wbbL*+ allele linked to a kanamycin resistance cassette from strain NR2528 was transduced into AV007 by P1-mediated transduction. Transductants were selected on LB + kan25 and confirmed via PCR.

EMF52(attHKHC859)

The *ftsH(V41G)* allele linked to the *leuU*-cat- *yhbX* marker was transduced from strain PR96 into strain HC533(attHKHC859) by P1-mediated transduction. Transductants were selected on LB + CM25 and the *ftsH(V41G)* allele was confirmed via sequencing.

EMF53(attHKHC859)
The *leuU*-cat-*yhbX* marker was transduced from strain PR90 into strain HC533(attHKHC859) by P1-mediated transduction. Transductants were selected on LB + CM25 and confirmed via PCR.

EMF212
MG1655 x P1(EMF211)
The kanamycin resistance cassette downstream of *wbbL(INS*) was transduced form EMF211 into MG1655 by P1-mediated transduction. Transductants were selected on LB + kan25 and confirmed by PCR.

EMF211
A kanamycin resistance cassette introduced to strain TB10 downstream of *wbbL(INS)* by lambda red recombineering. A PCR product amplified from pKD4 was generated using primers wbbL_kan_F (TCGCAACTTTGATCGAATTTCATCAGTTTTTCACCCGTAAGCGATTGTGTAGGCTGGAGC) and wbbL_kan_R (ATAAATAGCTTATCCATGCTTATATGCTTACGGCTTTATACTATTCCGAAGTTCCTATTC) and electroporated into strain TB10 following the protocol for strain DY329 described previously(1).

EMF214
The *wbbL*+ allele linked to a kanamycin resistance cassette from strain NR2528 was transduced into PR134 by P1-mediated transduction. Transductants were selected on M9 + CAA + glu + kan and confirmed via PCR.

PR35
The kanamycin resistance cassette in strain PR5 was cured using pCP20 as described previously (3).

EMF20
The *wbbL*+ allele linked to a kanamycin resistance cassette from strain NR2528 was transduced into PR35 by P1-mediated transduction. Transductants were selected on M9 + CAA + glu + kan and confirmed via PCR.

Plasmid Construction details

pPR112
The nativeRBS_lpxC insert was PCR amplified from E. coli K12 genomic DNA using forward primer LpxC_nativeRBS_XbaI5' (CCCCTCTAGATAATTTGGCGAGATAATACGATGATC) and reverse primer lpxC_3'truncation_HindIII (TGATAAGCTTATTAAGGCGCTTTGAAGGCCAACGG) resulting in an amplified PCR product of the nativeRBS and coding sequencing of lpxC lacking the 5 terminal amino acids. Primers contain xbaI and hindIII restriction sites, respectively. The PCR fragment was cloned into empty vector pPR66 using restriction enzymes xbaI and hindIII.

pPR115

pPR112 mutated with quickchange mutagenesis using primer lpxC_quickchange_H265A (TACCGCTTATAAATCCGGTGCTGCACTGAATAACAAACTG)

pEMF51

The *nativeRBS_fabZ(L85P)* insert was generated by amplifying the *fabZ* locus from strain EMF63 using primers xbaI_fabZ_F (ATCCTCTAGATGTCGTTTCTTATATTTTGACAGGAAGAG) and hindIII_fabZ_R (TACCAAGCTTTCAGGCCTCCCGGCTACG). This PCR product was digested with restriction enzymes xbaI and hindIII and ligated into pNP140.

pEMF137

pEMF51 and pPR66 were digested with restriction enzymes xbaI and hindIII-HF. The *nativeRBS_fabZ(L85P)* insert from pEMF51 was ligated into the pPR66 vector.

pEMF112

The plasmids pNP146 and pPR111 were digested with restriction enzymes xbaI and hindIII-HF. The pNP146 vector backbone was ligated with the *nativeRBS_lpxC* insert from pPR111

pEMF131

A PCR product was generated by amplifying the *nativeRBS_ pbp2(L61R)rodA* insert from pPR122 using primers pEMF131_F (CAAATCTAGATAAGGGAGCTTTGAGTAG) and pEMF131_R (TGATAAGCTTATGCGCACCTCTTACACGCTTTTC). The resulting PCR product was digested with restriction enzymes xbaI and hindIII-HF and ligated into the vector backbone of pNP146.

pPR122

A PCR product was generated by amplifying the *pbp2(L61R)* allele from genomic DNA from strain PR39 using primers XbaI-pbpA (GCTATCTAGATAAGGGAGCTTTGAGTAGAAAACG) and HindIII-pbpA (GCTAAAGCTTTTTATTCGGATTATCCGTCATG). This PCR product was digested with restriction enzymes xbaI and hindIII and ligated into the vector backbone of pHC857.

pEMF130

wbbL amplified from gDNA from strain AAY1using prmers wbbL XbaI-RBS-NdeI5': (TCTAGATTAAGAAGGAGATATACATATGGTATATATAATAATCGTTTCCCACGG) and wbbL_HindIII_Rev (AAGCTTTTACGGGTGAAAAACTGATGAAATTCGATCAAAGTTGCG). The resulting PCR product (xbaI-artificialRBS-wbbL) was cloned into vector pNP146 using restriction enzymes xbaI and hindIII.

pEMF134

lacZ was amplified from MG1655 gDNA using primers xbaI_strongRBS_lacZ (ATCCTCTAGACTTTAAGAAGGAGATATACCATGACCATGATTACGGATTCACTGG) and hindIII_lacZ_R (TGATAAGCTTATTATTTTTGACACCAGACCAACTGGTAATG). The resulting PCR product (xbaI-artificialRBS-lacZ) was cloned into vector pNP146 using restriction enzymes xbaI and hindIII. *The artificialRBS indicates the RBS of the F10 gene from T7 bacteriophage

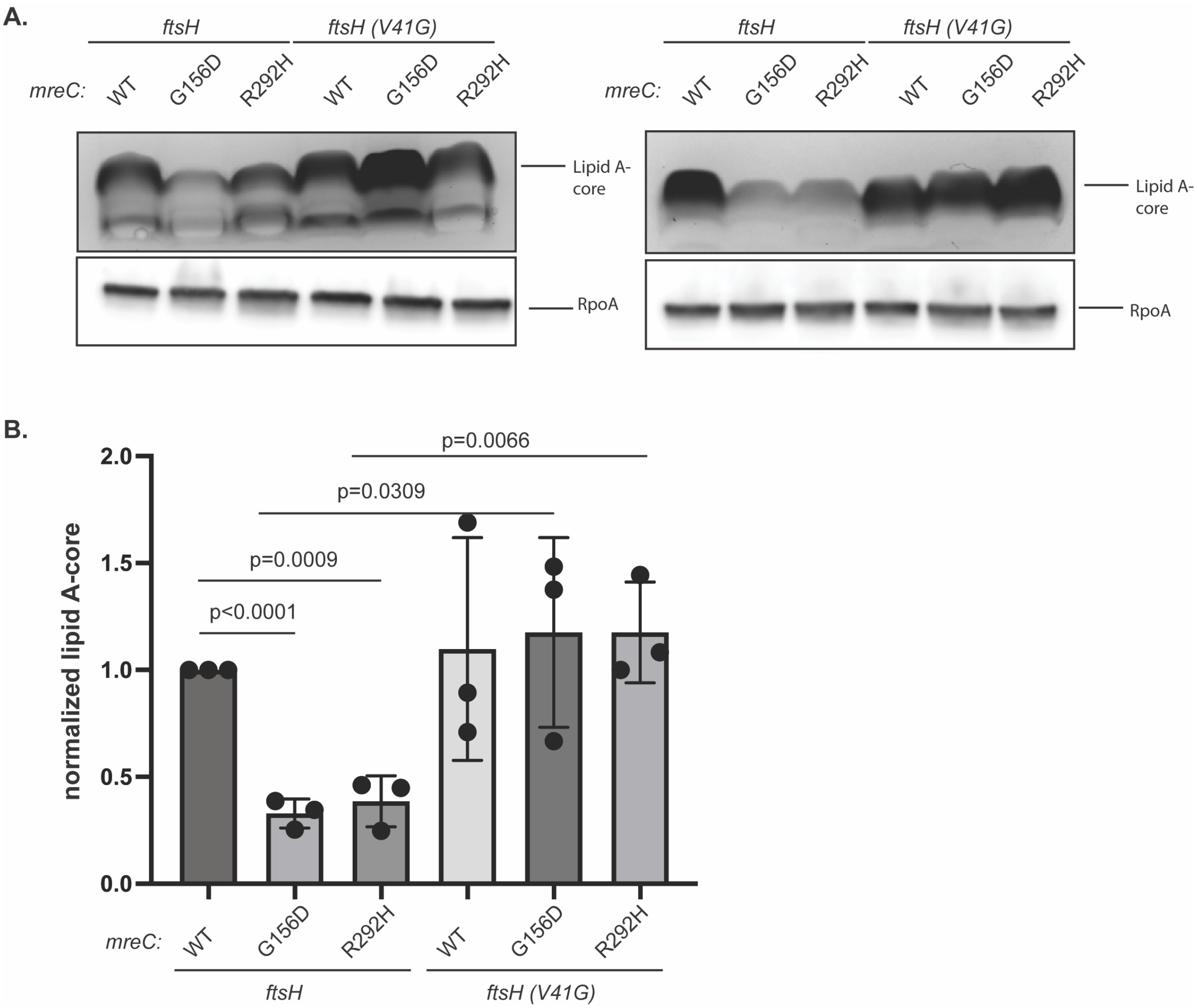

SI Figure 1: Quantification of lipid A-core levels in WT and *mreC* mutants with *ftsH(WT)* and *ftsH(V41G)* alleles. A. Replicates 2 and 3 of the lipid A-core silver stain corresponding to **Fig. 2D. B.** Lipid A-core levels were determined by densitometry using FIJI and plotted in Graphpad Prism. Quantification includes three biological replicates. Statistical significance determined by unpaired t test. We note that in order to visualize the low-abundant lipid A-core bands for *mreC(R292H)* and *mreC(G156D)*, the other bands were overexposed, which may affect the quantification and underestimate the difference between WT and mutant cells.

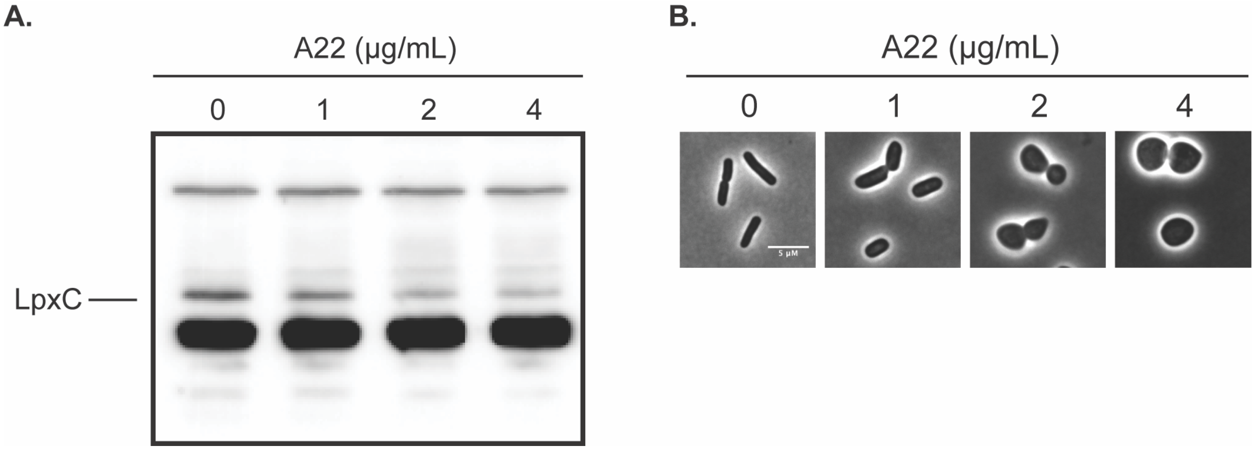

SI Figure 2: Treatment with A22 decreases LpxC levels. A. Immunoblot of LpxC levels in MG1655 cells treated with A22. MG1655 cells were cultured in LB overnight and then diluted to OD_{600}= 0.025 in LB. Cells were incubated at 37˚C until OD_{600} = 0.3 and then diluted to OD_{600} = 0.01 in LB + A22. Cells were cultured at 37˚C until OD_{600}= 0.3-0.4. **B.** Micrographs of MG1655 cells treated with A22. Scale bar = 5 μM.

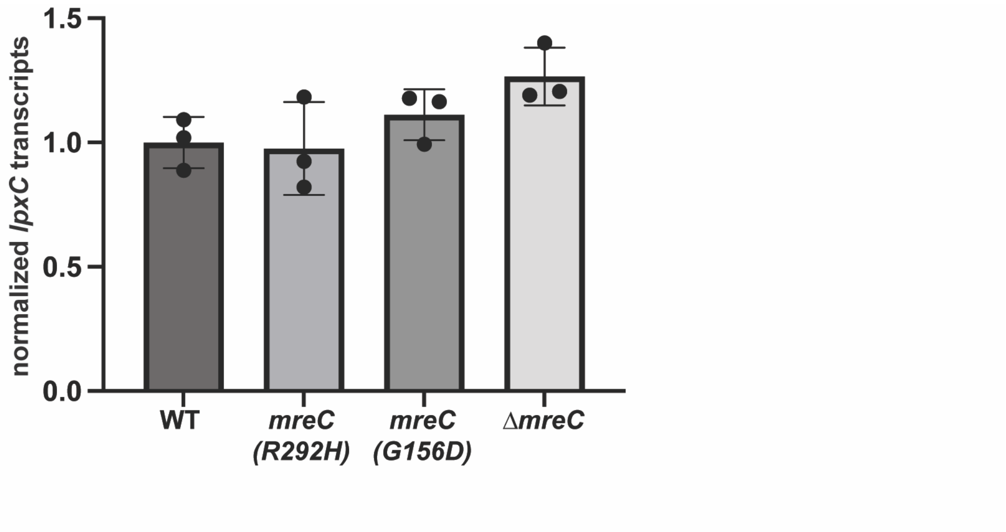

	P value	FDR
WT vs. *mreC(R292H)*	0.86852005	0.93694044
WT vs. *mreC(G156D)*	0.47453174	0.75311286
WT vs. Δ*mreC*	0.12828036	0.26425255

SI Figure 3: Transcript levels of *lpxC* in *mreC* mutants determined by RNAseq analysis. WT (HC555), *mreC(R292H)* (PR5), *mreC(G156D)* (PR30), and Δ*mreC* (EMF150) cells were cultured in M9 + CAA + glu for 24 hours at 30˚C. Cultures were then diluted to OD_{600}=0.05 in M9 + CAA + glu and incubated at 30˚C until OD_{600} = ~0.3. Cells were gently pelleted and resuspend in LB to a final OD = 0.025. Cultures were incubated at 37˚C until OD_{600} = 0.3-0.4. Cells were pelleted and pellets were stored at -80˚C and then analyzed via RNAseq by SeqCenter (Materials and Methods). Data from three biological replicates was plotted using Graphpad Prism. FDR = false discovery rate.

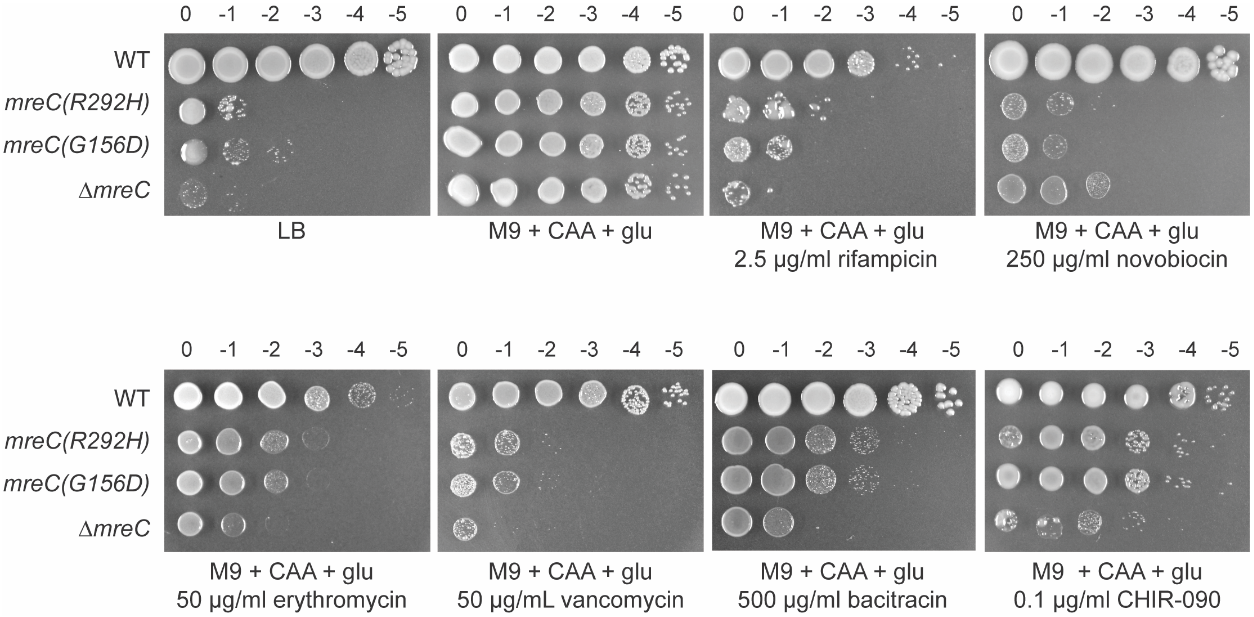

SI Figure 4: Antibiotic sensitivity of *mreC* mutants. WT (HC555), *mreC(R292H)* (PR5), *mreC(G156D)* (PR30), and Δ*mreC* (EMF150) cells were grown overnight in M9 + CAA + glu at 30˚C. Cultures were normalized to an OD_{600}=1, serially diluted, and spotted on the indicated plates. The plates were incubated at 30˚C. The LB plate was incubated for 24 hours and the M9 plates were incubated for 48 hours.

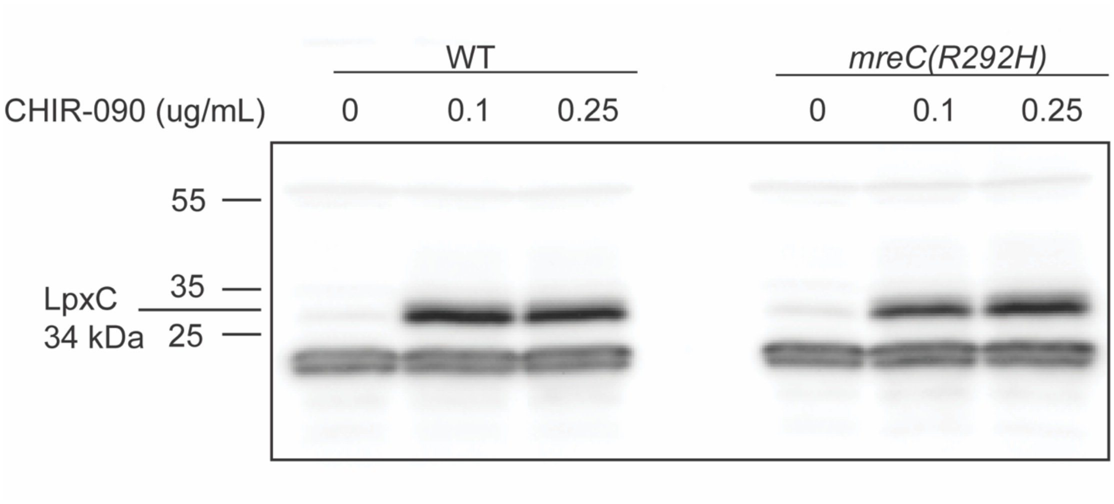

SI Figure 5: ***mreC(R292H)*** **cells homeostatically regulate levels of LpxC in response to LpxC inhibitor CHIR-090.** Immunoblot of LpxC levels in WT (HC555) and *mreC(R292H)* (PR5) cells treated with CHIR-090. Cells were grown for 24 hours at 30˚C in M9 + CAA + glu and then back diluted to OD_{600} = 0.05 in M9 + CAA + glu and incubated at 30˚C until OD_{600} = 0.4. Cells were gently pelleted and resuspended in LB and grown for one hour at 37˚C. CHIR-090 or DMSO was added to the cultures at the indicated concentrations. Cells were incubated for an additional hour at 37˚C before cell lysates were harvested for western blot.

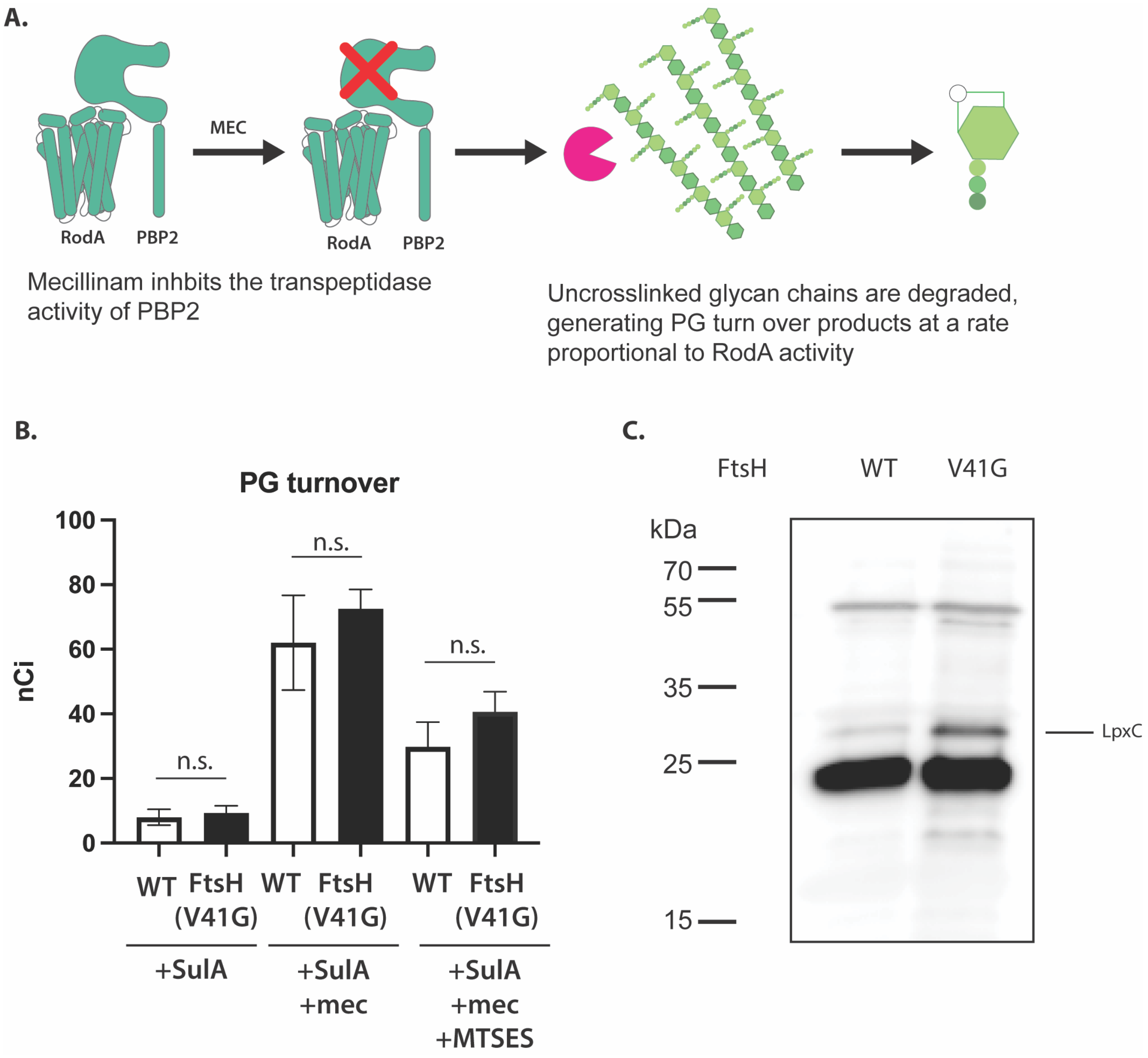

SI Figure 6: *ftsH(V41G)* does not increase PG synthesis by the Rod complex. A. Schematic of the generation of peptidoglycan turnover products (adapted from Rohs et al. 2018(4)). Glycan chains are polymerized by the glycosyltransferase RodA and cross linked into the cell wall matrix by PBP2. Mecillinam blocks the transpeptidases activity of PBP2, leading to the accumulation of uncrosslinked glycan polymers, which are then degraded, generating PG turnover products. These products include a radiolabeled mDAP residue, allowing for detection via HPLC and in-line scintillation counting. **B.** The amount of PG turnover products in WT and FtsH(V41G) cells. SulA blocks divisome activity and MTSES blocks PG synthesis by class A PBPs. Statistical significance was determined using an Unpaired t-test (n.s. indicates not significant). **C.** Immunoblot of LpxC in strains used for radiolabeling assay (see materials and methods).

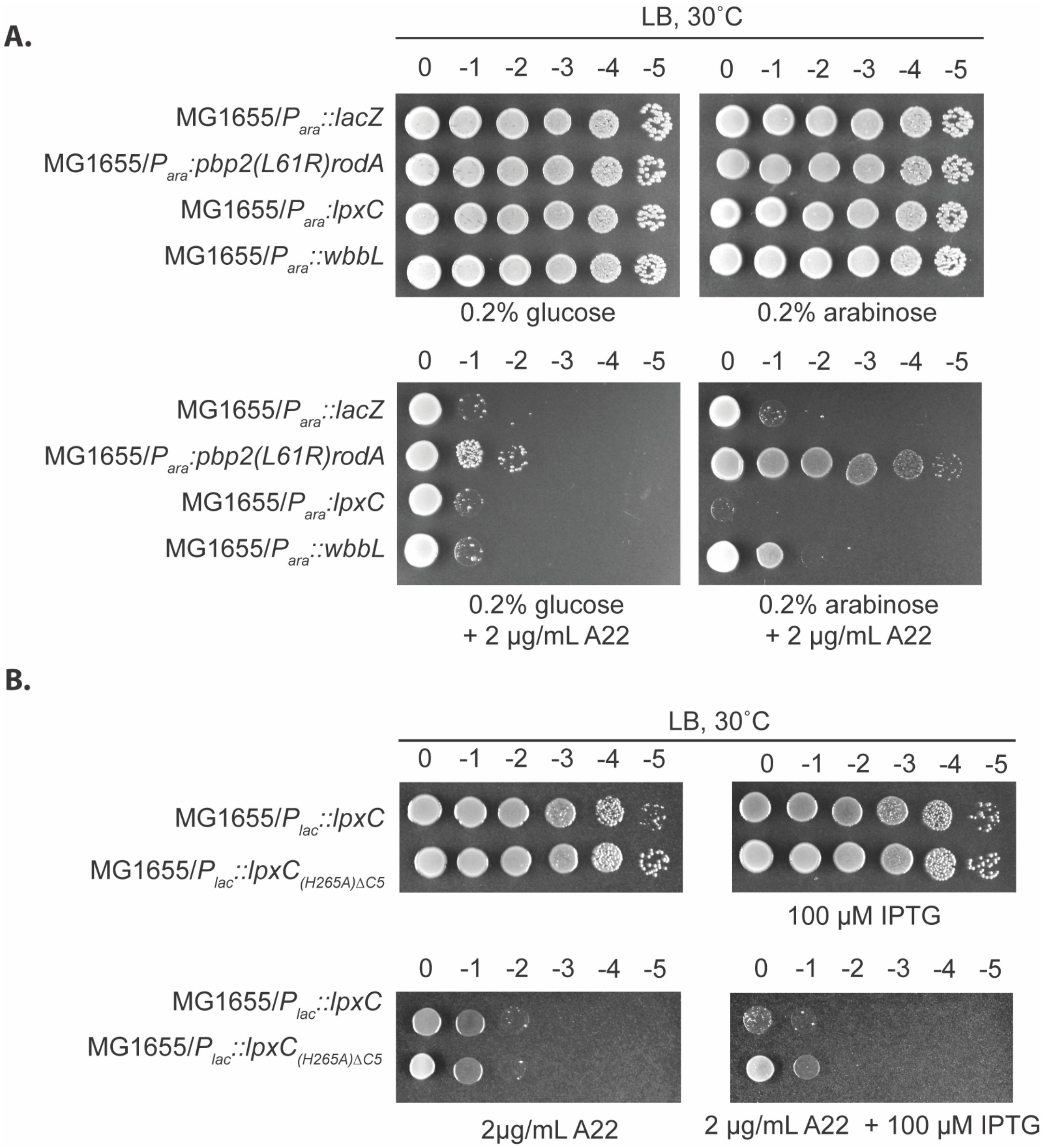

SI Figure 7: Overexpressing *lpxC* or *wbbL* does not confer A22 resistance. A. MG1655 cells harboring arabinose-inducible plasmids expressing *lacZ* (pEMF134), *pbp2(L61R)rodA* (pEMF131), *lpxC* (pEMF112), or *wbbL* (pEMF130) were grown overnight in LB. Cultures were normalized to an OD_{600}=1, serially diluted, and spotted on LB + 0.2% glucose or 0.2% arabinose plates with and without 2 µg/mL A22. Plates were incubated at 30˚C for 24 hours. **B**. MG1655 cells harboring an IPTG-inducible plasmid expressing *lpxC* (pPR111) or *lpxC(H265A)ΔC5* (pPR115) were grown o/n at 30˚C in LB. Cultures were normalized to an OD_{600}=1, serially diluted, and spotted on LB plates with and without 100 µM IPTG and 2 µg/mL A22. Plates were incubated at 30˚C for 24 hours.

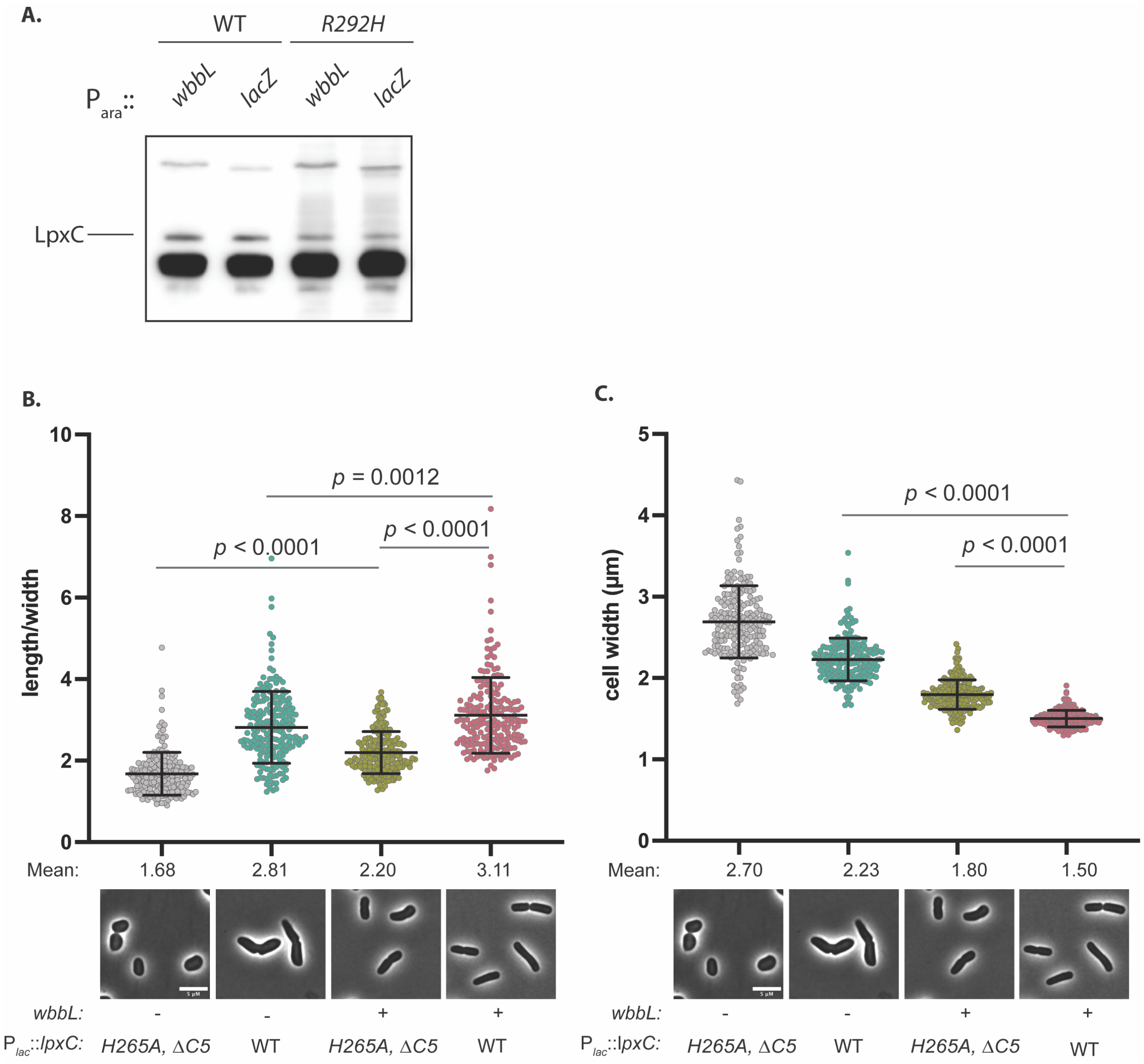

SI Figure 8: Increased LPS synthesis and O-antigen both contribute to restoration of rod shape in *mreC(R292H)* mutants*.* A. Immunoblot of LpxC levels in WT (HC555) and *mreC(R292H)* (PR5) expressing *wbbL* (pEMF130) or *lacZ* (pEMF134). Cells were grown as described in **Fig. 4C. B.** The aspect ratio and **C.** cell width of *mreC(R292H)* (PR35) or *mreC(R292H)* wbbL+ (EMF20) cells expressing WT *lpxC* (pPR111) or *lpxC(H265A)ΔC5* (pPR115). Cells were cultured as described in **Fig. 3.**

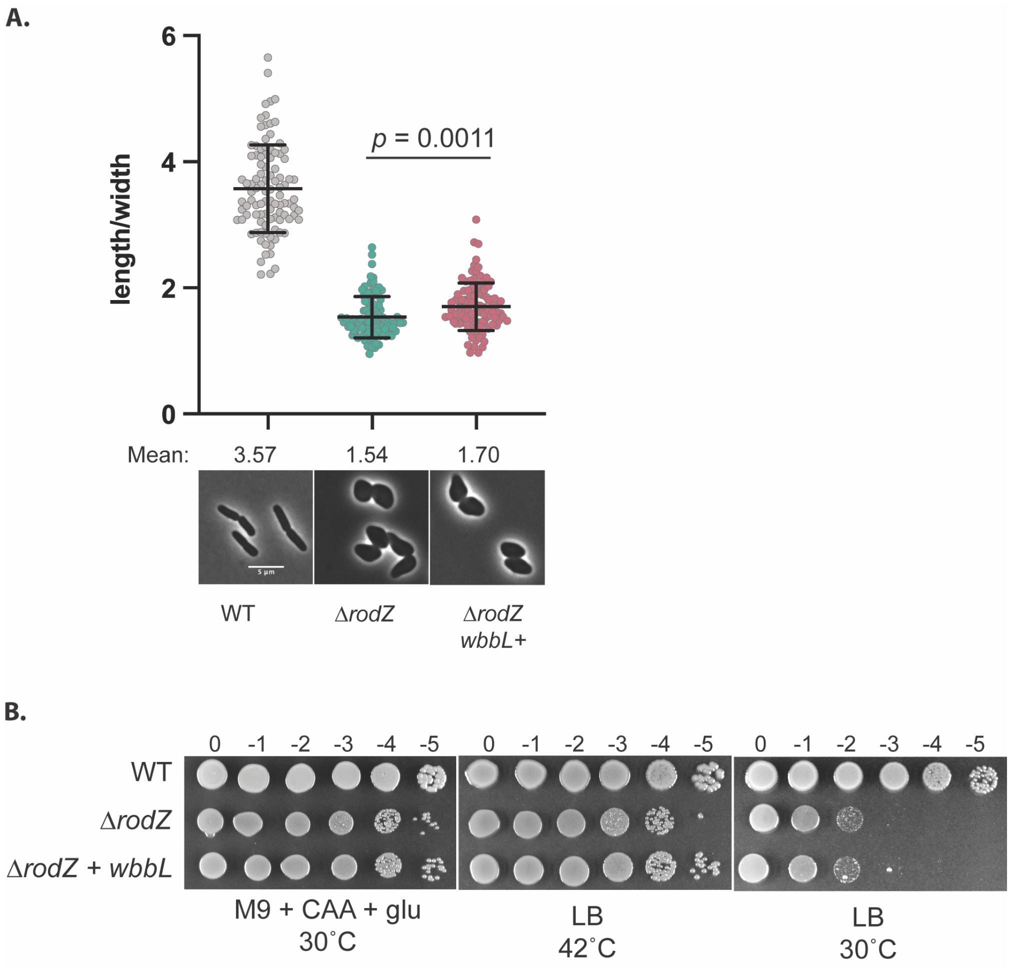

SI Figure 9: The overexpression of *wbbL* does not ameliorate the growth or shape defects of Δ*rodZ* cells. A. WT (EMF212), Δ*rodZ* (PR134), and Δ*rodZ wbbL*+ (EMF214) were cultures O/N in LB at 37˚C. Cultures were diluted at a ratio of 1:200 in LB and grown at 30˚C until OD_{600} = 0.3. Cells were then fixed and imaged. Aspect ratios were analyzed using the FIJI plugin MicrobeJ (5). Scale bar = 5 µm. n= 100 cells per group. Statistical significance determined using an Unpaired t test with Welch's correction (not assuming equal SDs). **B.** The strains listed in (A) were cultured in LB at 37˚C overnight. Cultures were normalized to OD_{600}=1, serially diluted, and spotted on M9 + CAA + glu or LB plates. LB plates were incubated for 16 hours and M9 + CAA + glu plates were incubated for 48 hours.

SI Movie 1: Time lapse of MreB in *wbbL(INS)* (AV007) and *wbbL (+)* (EMF210) cells expressing *mreC(R292H)D* (pMS9) described in Fig. 5. Three-minute timelapse series with an acquisition frame rate of 3s were recorded to capture MreB dynamics. TOP: ^{SW}*mreB-mNeon* overlayed over a single-frame phase contrast reference image. BOTTOM: Examples of MreB tracks identified using TrackMate (6, 7). Scale Bar = 2 μM.

Table S1: Suppressors of *mreC(R292H)* and *mreC(G156D)*

Suppressor	Background	Selection strategy	description
ftsH(V41G)	*mreC(G156D)*	Spontaneous suppressors, LB + 1% SDS at 30˚C	Inner membrane zinc-dependent metalloprotease that regulates the degradation of UDP-3-O-acyl-N-acetylglucosamine deacetylase (LpxC)(8, 9), the enzyme that catalyzes the first committed step in LPS synthesis (10, 11).
ftsH(F37V)	*mreC(R292H)*	Spontaneous suppressors, LB at 30˚C	
lapB(Δ379-389)	*mreC(G156D)*	Spontaneous suppressors, LB + 1% SDS at 30˚C	Lipopolysaccharide assembly protein B, mediates LpxC degradation by FtsH (12-14)

Table S2: Strains used in this study

Strain	Genotype[a]	Source/Reference[b]
AAY1	MG1655 *ΔlacIZYA<>frt wbbL+::kan*	(15)
AV007	MG1655 *mreB'-mNeon-'mreB ΔyhdE<> frt*	This study
Dh5a(lpir)	*F- hsdR17 deoR recA1 endA1 phoA supE44 thi-1 gyrA96 relA1 Δ(lacZYA-argF)U169 Ø80dlacZΔM15 λpir*	Laboratory strain
EMF20	MG1655 *mreC(R292H) yrdE-FRT* wbbL+::kan	This study, PR35 X P1(NR2528)(15)
EMF150	MG1655 *ΔmreC::kan*	This study, MG1655 X P1(MT4/pTB63)
EMF196	MG1655 *leuU-cat-yhbX yrdE-kan*	This study, PR103 x P1(HC555)
EMF197	MG1655 *leuU-cat-yhbX yrdE-kan mreC(G156D)*	This study, PR103 x P1(PR30/pTB63)
EMF199	MG1655 *leuU-cat-yhbX ftsH(V41G) yrdE-kan*	This study, PR104 x P1(HC555)
EMF210	MG1655 *mreB'-mNeon-'mreB ΔyhdE<> frt wbbL+::kan*	This study, AV007 X P1(NR2528)(15)
EMF211	TB10 *wbbL(INS)::kan*	This study
EMF212	MG1655 *wbbL(INS)::kan*	This study, MG1655 x P1(EMF211)
EMF214	MG1655 *ΔrodZ::cat wbbL+::kan*	This study, PR134 X P1(NR2528)(15)
EMF52(attHKHC859)	MG1655 *ΔlysA<>FRT ΔpbpC<>FRT ΔmtgA<>FRT ΔampD<>FRT mrcB(S247C) mrcA<>FRT leuU-cat-yhbX ftsH(V41G)*	This study, HC533(attHKHC859) X P1(PR96)
EMF53(attHKpHC859)	MG1655 *ΔlysA<>FRT ΔpbpC<>FRT ΔmtgA<>FRT ΔampD<>FRT mrcB(S247C) mrcA<>FRT leuU-cat-yhbX*	This study, HC533(attHKHC859)(16) X P1(PR90)

EMF63	MG1655 *fabZ(sfhC21)*	This study, EMF61 X P1(AR3289)(8)
HC555	MG1655 *yrdE-kan*	(4)
JAB593	MG1655 *mreB'-mNeon-'mreB ΔyhdE::cat*	(4)
MG1655	rph1 IvG rfb-50	(17)
MT4/pTB63	MG1655 *ΔlacIZYA<>frt mreC::kan*	(4)
PR103	MG1655 *leuU-cat-yhbX*	This study,MG1655 x P1(PR90)
PR104	MG1655 *leuU-cat-yhbX ftsH(V41G)*	This study, MG1655 X P1(PR96)
PR109	MG1655 *leuU-cat-yhbX yrdE-kan mreC(R292H)*	This study, PR103 x P1(PR5/pTB63)
PR110	MG1655 *leuU-cat-yhbX ftsH(V41G) yrdE-kan mreC(R292H)*	This study, PR104 X P1(PR5/pTB63)
PR111	MG1655 *leuU-cat-yhbX ftsH(V41G)yrdE-Kan mreC(G156D)*	This study, PR104 X P1(PR30/pTB63)
PR134	MG1655 *ΔrodZ::cat*	(4)
PR30	MG1655 *mreC(G156D) yrdE-kan*	(4, 18)
PR39	MG1655 *mreC(R292H) pbp2(L61R) yrdE-kan*	(4)
PR5	MG1655 *mreC(R292H) yrdE-kan*	(4, 18)
PR35	MG1655 *mreC(R292H) yrdE-FRT*	This study
PR82	MG1655 *mreC(R292H) yrdE-kan ftsH(F37V)*	This study
PR86	MG1655 *mreC(G156D) yrdE-kan lapB(Δ379-389)*	This study
PR88	MG1655 *mreC(G156D) yrdE-kan ftsH(V41G)*	This study
PR90	TB10 *leuU-cat-yhbX*	This study
PR96	MG1655 *leuU-cat-yhbX ftsH(V41G) yrdE-kan mreC(G156D)*	This study

TB10	MG1655 *λΔcro-bio nad::Tn10*	(19)
TB28	MG1655 *ΔlacIZYA::frt*	(20)

[a] The kanamycin resistance cassette (*kan*) and chlorophenicol resistance cassette (cat) are flanked by frt sequences for removal by FLP recombinase

[b] Strains generate by P1 transduction are described as follows: recipient strain X P1(donor strain) (See supplementary text for details)

Table S3: Plasmids used in this study

Plasmid	Relevant features[a]	Origin	Reference/source
pcp20	CM^{R}, Amp^{R}, FLP+, lambda cI857+	pSC101	(3)
pEMF112	Tet^{R}, P_{ara}::*nativeRBS_lpxC*	pBR/colE1	This study
pEMF130	Tet^{R} , P_{ara}::*artificalRBS_wbbL*	pBR/colE1	This study
pEMF131	Tet^{R}, P_{ara}::*nativeRBS_ pbp2(L61R)rodA*	pBR/colE1	This study
pEMF134	Tet^{R}, P_{ara}::*artificalRBS_lacZ*	pBR/colE1	This study
pEMF137	CM^{R}, P_{lac}:: *nativeRBS_fabZ(L85P)*	pBR/colE1	This study
pEMF51	Tet^{R}, P_{ara}:: *nativeRBS_fabZ(L85P)*	pACYC	This study
pHC857	CM^{R}, P_{lac}::*nativeRBS_pbpA-rodA*	pBR/colE1	(16)
pHC859	Tet^{R} , P_{tac}::*sulA*	R6K	(16)
pKD46	Amp^{R}, P_{ara}::lambda red genes for recombineering	pSC101	(2)
pMS9	CM^{R}, Pl_{ac}:*nativeRBS_mreC(R292H)mreD*	pBR/colE1	(18)
pNP140	Tet^{R}, P_{ara}:: *sulA*	pACYC	This study
pNP146	Tet^{R}, P_{ara}::*sulA*	pBR/colE1	(21)
pPR111	CM^{R}, P_{lac}::*nativeRBS_lpxC*	pBR/colE1	(22)
pPR112	CM^{R}, P_{lac}::*nativeRBS_lpxC∆C5*	pBR/colE1	This study
pPR115	CM^{R}, P_{lac}::*nativeRBS (H265A)_lpxC*	pBR/colE1	This study
pPR122	CM^{R}, P_{lac}::*nativeRBS_pbpA(L61R)rodA*	pBR/colE1	This study
pPR66	CM^{R}, P_{lac}::empty	pBR/colE1	(22)
pTB63	Tet^{R} , P_{native}::*ftsQAZ*	pSC101	(23)

[a] P_{lac} and Pt_{ac} refer to the lactose promoters and P_{ara} refers to the arabinose promoter. The artificialRBS indicates the RBS of the $\Phi 10$ gene from T7 bacteriophage.

Table S4: Primers used in this study

Primer name	Sequence	Strain/plasmid
leuU-yhbX_P2_F	ACCTTGAAACGATGGTGCCGGTACGCCTTAG TTATAAATTCATATGAATATCCTCCTTAG	PR90
leuU-yhbX_P1_R	TTGACACAATAAAGTGCCAATTATGTCAGTAG AAGGGAAAGTGTAGGCTGGAGCTGCTTC	PR90
LpxC_nativeRBS_XbaI5	CCCCTCTAGATAATTTGGCGAGATAATACGAT GATC	pPR111
hindIII_fabZ_R	TACCAAGCTTTCAGGCCTCCCGGCTACG	pEMF51
hindIII_lacZ_R	TGATAAGCTTATTATTTTTGACACCAGACCAA CTGGTAATG	pEMF134
HindIII-pbpA	GCTAAAGCTTTTTATTCGGATTATCCGTCATG	pPR122
lpxC_3'truncation_HindIII	TGATAAGCTTATTAAGGCGCTTTGAAGGCCAA CGG	pPR112
lpxC_quickchange_H265A	TACCGCTTATAAATCCGGTGCTGCACTGAATA ACAAACTG	pPR115
lpxC_R_HindIII	TGATAAGCTTATTATGCCAGTACAGCTGAAGG CGC	pPR111
pEMF131_F	CAAATCTAGATAAGGGAGCTTTGAGTAG	pEMF131
pEMF131_R	TGATAAGCTTATGCGCACCTCTTACACGCTTT TC	pEMF131
wbbL XbaI-RBS-NdeI5'	TCTAGATTAAGAAGGAGATATACATATGGTAT ATATAATAATCGTTTCCCACGG	pEMF130
wbbL_HindIII_Rev	AAGCTTTTACGGGTGAAAAACTGATGAAATTC GATCAAAGTTGCG	pEMF130
wbbL_kan_F	TCGCAACTTTGATCGAATTTCATCAGTTTTTC ACCCGTAAGCGATTGTGTAGGCTGGAGC	EMF211
wbbL_kan_R	ATAAATAGCTTATCCATGCTTATATGCTTACG GCTTTATACTATTCCGAAGTTCCTATTC	EMF211
xbaI_fabZ_F	ATCCTCTAGATGTCGTTTCTTATATTTTGACA GGAAGAG	pEMF51
xbaI_strongRBS_lacZ	ATCCTCTAGACTTTAAGAAGGAGATATACCAT GACCATGATTACGGATTCACTGG	pEMF134

XbaI-pbpA	GCTATCTAGATAAGGGAGCTTTGAGTAGAAAACG	pPR122

SI references

1. D. Yu *et al.*, An efficient recombination system for chromosome engineering in Escherichia coli. *Proc. Natl. Acad. Sci. PNAS* **97**, 5978-5983 (2000).
2. K. A. Datsenko, B. L. Wanner, One-step inactivation of chromosomal genes in Escherichia coli K-12 using PCR products. *Proc. Natl. Acad. Sci. USA* **97**, 6640-6645 (2000).
3. P. P. Cherepanov, W. Wackernagel, Gene disruption in Escherichia coli: TcR and KmR cassettes with the option of Flp-catalyzed excision of the antibiotic-resistance determinant. *Gene* **158**, 9-14 (1995).
4. P. D. Rohs *et al.*, A central role for PBP2 in the activation of peptidoglycan polymerization by the bacterial cell elongation machinery. *PLoS Genet.* **14**, e1007726 (2018).
5. A. Ducret, E. M. Quardokus, Y. V. Brun, MicrobeJ, a tool for high throughput bacterial cell detection and quantitative analysis. *Nat. Microbiol.* **1**, 1-7 (2016).
6. J.-Y. Tinevez *et al.*, TrackMate: An open and extensible platform for single-particle tracking. *Methods* **115**, 80-90 (2017).
7. D. Ershov *et al.*, TrackMate 7: integrating state-of-the-art segmentation algorithms into tracking pipelines. *Nat. Methods*, 1-4 (2022).
8. T. Ogura *et al.*, Balanced biosynthesis of major membrane components through regulated degradation of the committed enzyme of lipid A biosynthesis by the AAA protease FtsH (HflB) in Escherichia coli. *Mol. Microbiol.* **31**, 833-844 (1999).
9. K. Ito, Y. Akiyama, Cellular functions, mechanism of action, and regulation of FtsH protease. *Annu. Rev. Microbiol.* **59**, 211-231 (2005).
10. M. S. Anderson, A. D. Robertson, I. Macher, C. R. Raetz, Biosynthesis of lipid A in Escherichia coli: identification of UDP-3-O-[(R)-3-hydroxymyristoyl]-. alpha.-D-glucosamine as a precursor of UDP-N2, O3-bis [(R)-3-hydroxymyristoyl]-. alpha.-D-glucosamine. *Biochem.* **27**, 1908-1917 (1988).
11. K. Young *et al.*, The envA permeability/cell division gene of Escherichia coli encodes the second enzyme of lipid A biosynthesis. UDP-3-O-(R-3-hydroxymyristoyl)-N-acetylglucosamine deacetylase. *J. Biol. Chem.* **270**, 30384-30391 (1995).
12. G. Klein, N. Kobylak, B. Lindner, A. Stupak, S. Raina, Assembly of lipopolysaccharide in Escherichia coli requires the essential LapB heat shock protein. *J. Biol. Chem.* **289**, 14829-14853 (2014).
13. S. Mahalakshmi, M. Sunayana, L. SaiSree, M. Reddy, yciM is an essential gene required for regulation of lipopolysaccharide synthesis in Escherichia coli. *Mol. Microbiol.* **91**, 145-157 (2014).
14. S. Shu, W. Mi, Regulatory mechanisms of lipopolysaccharide synthesis in Escherichia coli. *Nat. Commun.* **13**, 1-11 (2022).
15. L. T. Sham, S. Zheng, A. A. Yakhnina, A. C. Kruse, T. G. Bernhardt, Loss of specificity variants of WzxC suggest that substrate recognition is coupled with transporter opening in MOP-family flippases. *Mol. Microbiol.* **109**, 633-641 (2018).
16. H. Cho, T. Uehara, T. G. Bernhardt, Beta-lactam antibiotics induce a lethal malfunctioning of the bacterial cell wall synthesis machinery. *Cell* **159**, 1300-1311 (2014).

17. M. Guyer, R. R. Reed, J. Steitz, K. Low (1981) Identification of a sex-factor-affinity site in E. coli as γδ. in *Cold Spring Harbor symposia on quantitative biology* (Cold Spring Harbor Laboratory Press), pp 135-140.
18. P. D. Rohs *et al.*, Identification of potential regulatory domains within the MreC and MreD components of the cell elongation machinery. *J. Bacteriol.* **203**, e00493-00420 (2021).
19. J. E. Johnson, L. L. Lackner, C. A. Hale, P. A. De Boer, ZipA is required for targeting of DMinC/DicB, but not DMinC/MinD, complexes to septal ring assemblies in Escherichia coli. *J. Bacteriol.* **186**, 2418-2429 (2004).
20. T. G. Bernhardt, P. A. De Boer, Screening for synthetic lethal mutants in Escherichia coli and identification of EnvC (YibP) as a periplasmic septal ring factor with murein hydrolase activity. *Mol. Microbiol.* **52**, 1255-1269 (2004).
21. J. A. Buss, N. T. Peters, J. Xiao, T. G. Bernhardt, ZapA and ZapB form an FtsZ-independent structure at midcell. *Mol. Microbiol.* **104**, 652-663 (2017).
22. E. M. Fivenson, T. G. Bernhardt, An Essential Membrane Protein Modulates the Proteolysis of LpxC to Control Lipopolysaccharide Synthesis in Escherichia coli. *mBio* **11**, e00939-00920 (2020).
23. F. O. Bendezú, P. A. De Boer, Conditional lethality, division defects, membrane involution, and endocytosis in mre and mrd shape mutants of Escherichia coli. *J. Bacteriol.* **190**, 1792-1811 (2008).
